# Treating a systemic disease as a local one: failure points of current pancreatic cancer management and a multimodal, biology-guided framework for redesign

**DOI:** 10.3389/fonc.2026.1941793

**Published:** 2026-10-12

**Authors:** Patrick E. Sewell

**Affiliations:** Triple Helix Science, Santa Ana, CA, United States

**Keywords:** cryoablation, gene therapy, *in-situ* vaccination, neoantigen vaccine, occult metastasis, pancreatic ductal adenocarcinoma, resectability, tumor microenvironment

## Abstract

Pancreatic ductal adenocarcinoma remains among the most lethal solid tumors, with 5-year survival near 13% despite decades of incremental change, and current management continues to be organized around anatomic resectability and a chemotherapy backbone applied largely without regard to tumor biology. This article advances the thesis that the dominant paradigm underperforms because it treats a systemic, molecularly convergent, multi-compartment disease as though it were a local one. We first summarize the real advances of the past decade—in surgery, systemic therapy, molecular profiling, and immunotherapy—and then identify five interlocking failure points that persist despite them: (i) selection by vessel anatomy, judged on imaging criteria that are read inconsistently and that classify inaccurately even where read consistently, and applied to splanchnic veins without accounting for their baseline variability and tumor-associated thrombosis; (ii) occult metastasis and the resulting limits of local cure for the systemic majority, evidenced by the gap between complete resection and durable survival and by early distant recurrence; (iii) the addition of radiotherapy, which improves local control without extending survival; (iv) chemotherapy chosen without reference to a validated prognostic classical/basal-like distinction, although prospective subtype-directed regimen selection has not yet improved outcomes in randomized testing; and (v) a sequential, non-integrated structure in which systemic and immune therapy are deferred and no definitive path exists for the roughly 80% who are not surgical candidates. We then propose a multimodal, biology-guided framework that answers each failure point, with every component graded on an explicit evidence scale: corrected tumor-suppressor gene therapy matched to lesion type, a systemic backbone combining renin-angiotensin system-pathway inhibition with immunogenically sequenced chemotherapy and personalized neoantigen vaccination, *in-situ* immune conversion of an accessible tumor by cryoablation with local priming and natural killer (NK) cell delivery, and front-end biological patient selection. The framework is conceptual rather than product-specific; we specify its rationale, its integration in time, a staged first-in-human development program, and the studies required to test it. The objective is to convert local disease into systemic benefit—a step the current paradigm is not designed to take.

## Introduction

The treatment of pancreatic ductal adenocarcinoma (PDAC) is built on an anatomical premise. Guidelines stratify newly diagnosed patients into resectable, borderline resectable, locally advanced, and metastatic categories defined chiefly by the relationship of the tumor to nearby arteries and veins on cross-sectional imaging, and this single anatomical judgment then governs the entire treatment course (1). Surgery is offered to those whose tumors are deemed anatomically removable and is described as the only potentially curative modality; chemotherapy is the backbone at every stage; radiotherapy is applied selectively; and molecular characterization, where performed, rarely changes first-line management outside the small breast cancer gene (BRCA) and mismatch-repair-deficient subgroups. The logic is intuitive and, for a genuinely localized cancer, sound.

The difficulty is that anatomic localization in PDAC is frequently biologically misleading: the disease is more often systemic than its radiographic appearance suggests, and a genuinely organ-confined biology is the exception rather than the rule. It behaves as a systemic disease from an early point in its natural history, converges molecularly on a small set of driver lesions, and is embedded in a physical and immunological microenvironment that defeats single-agent approaches (2–4). Judged against that biology, the anatomic paradigm commits the central therapeutic decision to a feature—vessel contact—that is only loosely related to whether the disease can actually be controlled, and it organizes care around removing a primary tumor in a disease whose lethality is overwhelmingly determined by disease that has already left it. The consequence is a treatment structure that cures a minority, harms many, and offers no coherent path to the majority.

This is not an argument against surgery, and not an argument that current care has stood still. Surgery cures a genuine minority whose disease is truly confined, and it remains the correct choice for that minority; the last decade has also produced measurable gains, reviewed below, that any fair account must credit. The argument is narrower and structural: that the organizing variable—anatomy—is the wrong one for most patients.

The paper is organized in two movements. The first is a critical anatomy of the current paradigm: after crediting recent advances, it establishes the disease’s systemic, convergent, multi-compartment structure, then examines five failure points in turn and shows that they share a single root. The second is constructive: it derives the design requirements that follow from the critique and proposes a multimodal, biology-guided framework that answers each failure point, presented as a testable hypothesis with its evidence grading, limitations, feasibility, and validation path made explicit. Throughout, we distinguish claims well supported by existing evidence, which dominate the critique, from the proposal itself, which we present explicitly as a hypothesis to be tested.

## What this article adds

Because the framework integrates several concepts that are individually familiar, it is worth stating at the outset which elements are genuinely new and which are adaptations of existing strategies, so the contribution is not overstated. Three ideas in the framework are adaptations: *in-situ* immune conversion of an ablated tumor, personalized neoantigen vaccination, and renin-angiotensin system (RAS)–pathway inhibition are each established lines of work that we integrate rather than originate, and we credit their sources directly. What is new is not any single component but the argument that binds them, and it has four parts. First, a unified diagnosis: the article treats five well-documented shortcomings of PDAC management as surface expressions of one error—treating a systemic, multi-compartment disease as a local one—rather than as independent problems. Second, there is an explicit design logic that derives the requirements of an adequate treatment from the structure of the disease and, in particular, the claim that the barriers are addressable only in a defined order. Third, the specific proposal to add corrected, lesion-type-matched tumor-suppressor gene therapy into the same accessible lesion, at the same session, as an immunogenic ablation, a combination that, to our knowledge, is absent from prior ablation-immunotherapy programs. Fourth, the reframing of front-end selection from anatomy to biology as the single most immediately testable and least agent-encumbered change the critique implies. The critique is intended to be the durable contribution; the framework is one concrete, falsifiable instantiation of the redesign the critique demands, and its value does not depend on every component proving correct.

## Recent advances in the management of pancreatic cancer

An honest critique must begin by crediting what has improved, both because objectivity requires it and because the residual failures are clearer against that background. The past decade has produced real, measurable gains on several fronts (5).

Surgery has become safer and more selective. Perioperative mortality at high-volume centers has fallen, minimally invasive pancreatectomy has matured, and the centralization of complex resection into higher-volume centers has improved outcomes; venous resection and reconstruction, once exceptional, are now a routine part of the surgical repertoire (6, 7). Systemic therapy has advanced from single-agent gemcitabine to multi-agent regimens that meaningfully extend survival: FOLFIRINOX (folinic acid, fluorouracil, irinotecan, and oxaliplatin) and gemcitabine/nab-paclitaxel, and more recently the combination chemotherapy regimen (NALIRIFOX) regimen, have raised response rates and median survival in the metastatic setting, and adjuvant modified fluorouracil, leucovorin, irinotecan, and oxaliplatin (mFOLFIRINOX) improved 5-year overall survival of resected patients from 31.4% to 43.2% relative to gemcitabine in the Partenariat de Recherche en Oncologie Digestive-24 (PRODIGE-24) trial (8–10). Molecular profiling has entered practice for a defined minority: BRCA and Partner and localizer of BRCA2 (PALB2) status now directs maintenance poly (ADP-ribose) polymerase inhibition, mismatch-repair deficiency directs checkpoint blockade, and rarer events [neurotrophic tyrosine receptor kinase and neuregulin 1 fusions, Kirsten rat sarcoma viral oncogene homolog (KRAS) G12C] are actionable where found. Most consequentially, the KRAS pathway—long considered undruggable and mutated in the overwhelming majority of PDAC—has become a therapeutic target: in the randomized RASolute 302 trial in previously treated metastatic disease, the RAS(ON) multi-selective inhibitor daraxonrasib approximately doubled median overall survival versus chemotherapy (11). Immunotherapy, though still ineffective for the checkpoint-refractory majority, has produced a credible proof of principle in the form of individualized mRNA neoantigen vaccines that induce durable T-cell responses correlated with delayed recurrence after resection (12, 13).

These advances also constrain the argument of this paper in specific ways, and three randomized results deserve explicit attention because they cut against intuitions the framework might otherwise invoke. First, intensifying neoadjuvant therapy has not been uniformly beneficial: in Nordic Pancreatic Cancer Trial-1 (NORPACT-1), neoadjuvant FOLFIRINOX for clearly resectable pancreatic head cancer did not improve survival and numerically favored upfront surgery (14), and in the Preoperative Chemoradiotherapy Versus Immediate Surgery for Resectable and Borderline Resectable Pancreatic Cancer-2 (PREOPANC-2) trial, neoadjuvant FOLFIRINOX and neoadjuvant gemcitabine-based chemoradiotherapy produced no difference in overall survival (15). Neoadjuvant sequencing is therefore valuable as a biological filter, as argued below, but is not by itself a survival lever, and the framework does not claim otherwise. Second, and most important for the argument that follows, the Pancreatic Adenocarcinoma Signature Stratification for Treatment-01 (PASS-01) trial directly tested whether transcriptomic subtype predicts differential chemotherapy benefit—the premise on which subtype-directed regimen selection rests—and did not confirm it. Overall survival favored gemcitabine/nab-paclitaxel over modified FOLFIRINOX (9.7 vs. 8.5 months; hazard ratio 1.57, 95% CI 1.08–2.28, *P* = .017), and no subtype prospectively identified a subgroup benefiting from FOLFIRINOX. In the classical subtype, an exploratory subgroup comparison—not a formal test of interaction—again favored gemcitabine/nab-paclitaxel (13.9 vs. 9.7 months, *P* = .047), the opposite of the retrospective expectation (16). This is a genuine constraint on Failure Point IV, and we address it directly there rather than around it: the classical/basal-like distinction remains prognostically real and biologically validated, but the specific claim that it should be used to choose between existing first-line regimens is, on current randomized evidence, unproven. The framework accordingly uses subtype for prognosis, risk stratification, and trial design rather than as a validated instrument for regimen switching. Recognizing these results does not weaken the central thesis; it sharpens it, because none of them rescues anatomic selection or converts local control into systemic benefit—the two problems the framework is built to address.

## The scale and structure of the problem

Any redesign must begin from the disease’s actual structure, because a therapy succeeds or fails according to which layer of it the therapy engages. PDAC presents four barriers simultaneously, and no single-compartment intervention crosses more than one.

### Epidemiology and stage distribution

The epidemiology is most honestly stated from the majority’s perspective. By the diagnostic criteria in current use, 85%–90% of patients present with disease already beyond anatomically resectable—roughly 30%–35% borderline or locally advanced, and about half overtly metastatic—leaving only 10%–15% deemed resectable (1, 17). Even with the best treatment available today, all-stage 5-year survival is near 13%, meaning that approximately 87% of patients are dead within 5 years of diagnosis (17).

The more revealing question is how many of the 10%–15% deemed resectable are actually cured by resection, and the answer is fewer than the label implies. In a Surveillance, Epidemiology, and End Results (SEER) cure-model analysis of 125,706 patients, the statistical cure fraction—the proportion whose mortality returns to that of the age- and sex-matched general population—was only 5.7% for the whole cohort; even among the 20.5% who underwent resection it was 25.2%, falling to 17.1% for those treated with upfront surgery (18). A multi-institutional analysis using a disease-free-survival cure model placed the resected cure fraction at 20.4% (19). By either estimate, of the small minority selected for resection by anatomic criteria, roughly three-quarters are not cured by the operation. This is not primarily a failure of surgical technique but of selection: the criteria identify tumors that look removable while admitting a large majority whose disease is already systemic—the problem developed under Failure Point I below.

The same reality explains the otherwise puzzling survival of “localized” disease. Stage-stratified 5-year survival is approximately 44% for localized, 17% for regional, and 3% for distant disease (17)—yet 44% is far below what a genuinely organ-confined cancer should yield, because anatomic localization and biological localization are not the same thing. PDAC disseminates extraordinarily early: in a lineage-tracing mouse model, tagged pancreatic epithelial cells underwent epithelial-to-mesenchymal transition, entered the bloodstream, and seeded the liver before frank malignancy could be detected histologically (20). A tumor radiographically confined to the pancreas has therefore, in a substantial fraction of cases, already released the cells that will become the metastases that kill the patient. “Localized” names the appearance of the primary, not the distribution of the disease (**Figure 1**).

**Figure 1:**
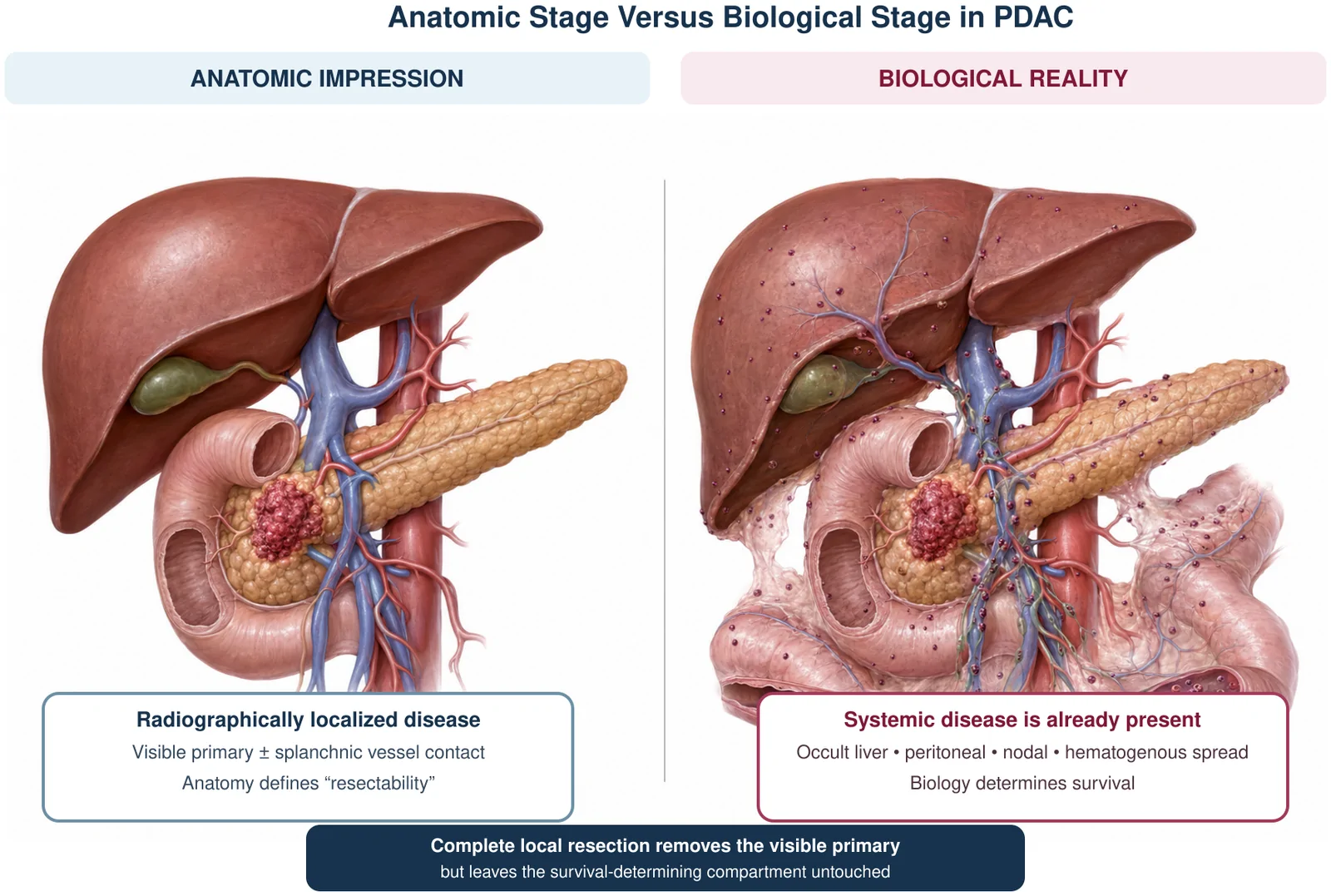
Anatomic stage versus biological stage in PDAC. Schematic contrasting the radiographic impression of localized disease (a single primary in contact with splanchnic vessels) with the biological reality—occult micrometastatic dissemination present at diagnosis in the majority, consistent with evidence that pancreatic cells enter the circulation and seed the liver before frank malignancy is histologically detectable (20). Complete local resection removes the visible primary while leaving the survival-determining compartment untouched, motivating biological rather than anatomic staging.

These figures compose a stark arithmetic worth stating as a single funnel (**Figure 2**). Of every 100 patients who present with PDAC, about 20 undergo resection (18); of those 20, roughly a quarter are cured; so the entire curative yield of modern management—surgery, chemotherapy, and radiation combined—is on the order of five to six cures per 100 patients. Cure is, moreover, almost entirely a property of the surgical pathway: it is concentrated in resected patients and augmented by perioperative chemotherapy (which raises the resected cure fraction from ~17% with upfront surgery to ~30% with neoadjuvant treatment (18)), whereas chemotherapy in unresectable or metastatic disease extends survival by months without curing. This is why all-stage 5-year survival (~13%) exceeds the cure fraction (~5.7%): the gap is not additional cures but patients alive at 5 years yet not cured, because statistical cure in PDAC is not reached for roughly a decade (17, 18). The currency of everything beyond surgery is time, not cure—a fact with consequences for both the biology and the economics of the disease, developed below.

**Figure 2:**
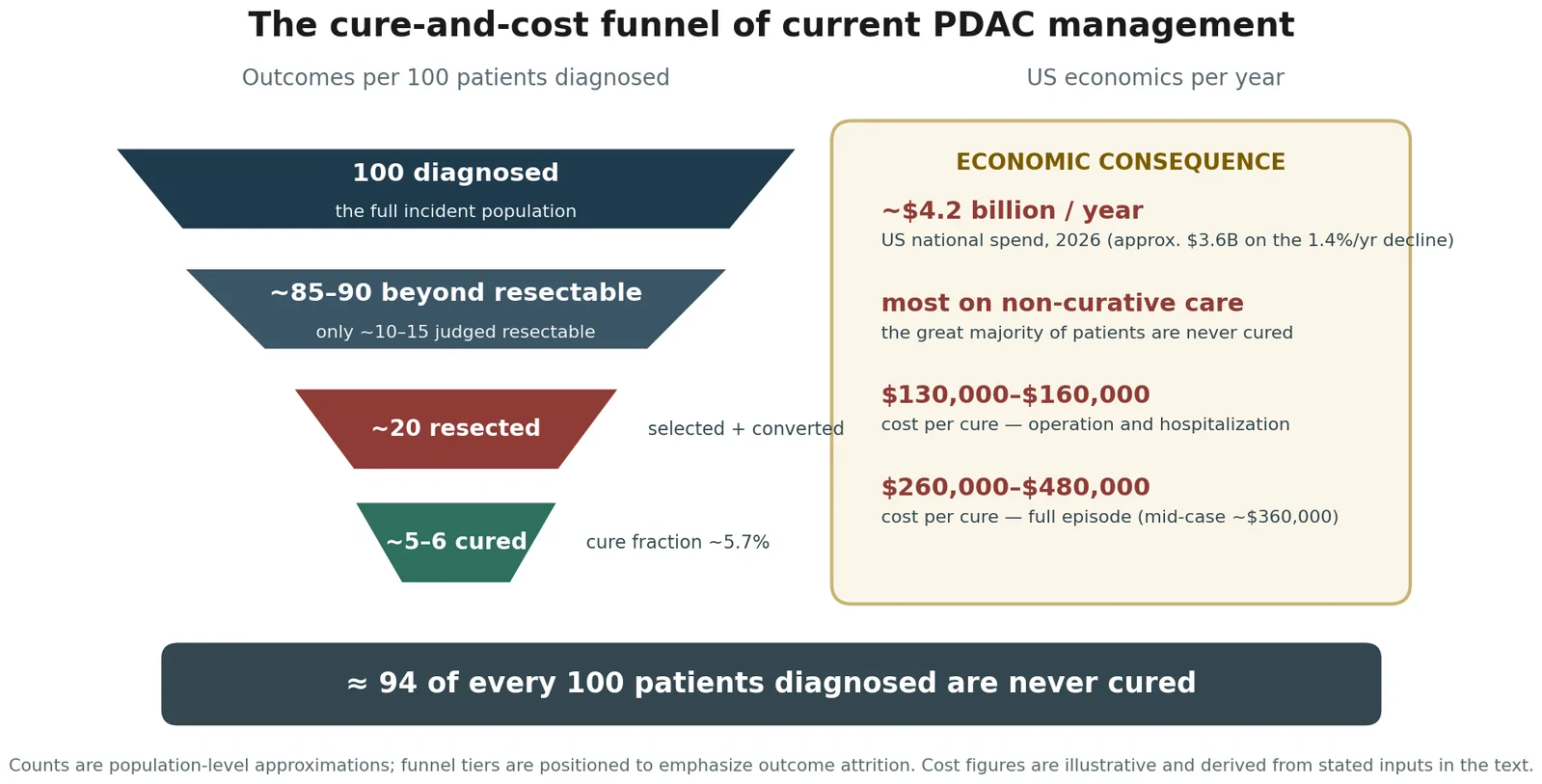
The cure-and-cost funnel of current management (per 100 patients). Attrition from diagnosis to cure: of every 100 patients diagnosed, roughly 85–90 already present beyond anatomically resectable, about 20 undergo resection (the resected fraction can exceed the fraction resectable at presentation because neoadjuvant therapy converts some borderline or locally advanced tumors), and only ~5–6 achieve statistical cure (18). The funnel is annotated with the system-level economics developed in the text: a US national spend on the order of $4.2 billion per year (2026), most of it on care that does not achieve cure, and a cost per actual cure of roughly $130,000–$160,000 (operation and hospitalization) to $260,000–$480,000 (full care episode).

### Molecular convergence

Genetically the disease is strikingly uniform. Activating KRAS mutations occur in roughly 90%–95% of tumors, and the principal tumor suppressors are inactivated at high frequency: TP53 in about 70%, most often by dominant-negative missense mutation; cyclin-dependent kinase inhibitor 2A (CDKN2A) in 50%–95%; and SMA- and MAD-related protein 4 (SMAD4) in about half (2, 3). This convergence is a liability of current therapy—none of these lesions is addressed in standard care—but an opportunity for a rationally designed one, because a small set of corrections addresses most tumors.

### Transcriptional subtypes

Superimposed on this genetic uniformity is a reproducible transcriptional split into a chemoresistant basal-like subtype and a more treatment-responsive classical subtype, distinguishable in practice by GATA binding protein 6 (GATA6) expression (21, 22). This distinction is prognostically validated and biologically real. It is important, however, to state its limits precisely: a randomized trial that prospectively used subtype to inform first-line regimen choice (PASS-01) did not show that subtype predicts differential benefit between the two standard multi-agent regimens (16). The distinction therefore carries prognostic and stratification value that current first-line selection still ignores, but it is not, on present evidence, a validated switch between existing regimens—a nuance we return to under Failure Point IV.

### A hostile physical and immune microenvironment

PDAC is a minority of malignant cells within a majority of stroma and suppressive immune cells. Heterogeneous populations of cancer-associated fibroblasts—myofibroblastic, inflammatory, and antigen-presenting subsets—build a stiff, hyaluronan-rich matrix that, combined with high interstitial fluid pressure, collapses tumor vasculature and limits drug delivery to a fraction of systemic levels (4, 23–25). This physical barrier is a principal reason that systemically active drugs underperform in PDAC relative to other adenocarcinomas, because the agent never reaches the malignant compartment at effective concentration. The immune microenvironment is “cold”: effector T cells are physically excluded and held off by C-X-C motif chemokine ligand 12/fibroblast activation protein signaling, myeloid-derived suppressor cells dominate, and fewer than 1% of tumors are mismatch-repair deficient, so checkpoint blockade largely fails (26, 27). Each layer defines a barrier that a single agent cannot cross, and together they explain why incremental monotherapy has not moved the disease.

Two features of this structure are decisive for design. First, the barriers are not alternatives but a stack: a therapy can correct a driver lesion and still fail because the stroma prevents delivery, or achieve delivery and still fail because the immune compartment cannot execute killing. Second, the barriers are addressable in a defined order—decompress the matrix to enable delivery, correct the drivers and release antigen, then recruit and license effectors—which is precisely the logic a multi-compartment program can exploit but a local-first, single-agent paradigm cannot. The structure of the disease already specifies the structure of an adequate treatment.

## Failure point I—selection by anatomy rather than biology

The first failure is that the pivotal treatment decision is made on the wrong variable and measured imperfectly. Resectability is defined by the degree of tumor contact with the superior mesenteric artery, celiac axis, common hepatic artery, and the superior mesenteric and portal veins on computed tomography (CT) (**Table 1**) (1). Several problems undermine this from the outset.

**Table 1:** The anatomic resectability criteria in current use, against which the treatment decision is made.

Category	Arterial contact (SMA, celiac axis, CHA)	Venous contact (SMV/PV)
Resectable	No arterial tumor contact	No contact, or ≤ 180°contact without vein contour irregularity
Borderline resectable	Contact ≤ 180° with SMA or celiac axis; contact with CHA without extension to the celiac axis/bifurcation	Contact > 180°, or ≤ 180° with contour irregularity or thrombosis, with vein suitable proximally and distally for reconstruction
Locally advanced (unresectable)	Contact > 180° with SMA or celiac axis; aortic involvement	Unreconstructable SMV/PV owing to tumor involvement or occlusion

Criteria per National Comprehensive Cancer Network guidelines (1). SMA, superior mesenteric artery; CHA, common hepatic artery; SMV, superior mesenteric vein; PV, portal vein.

### The criteria are imperfectly reproducible and, where reproducible, inaccurate

The anatomic categorization has two independent weaknesses: it is read inconsistently, and even where it is read consistently it classifies inaccurately. On the first, in a multi-reader study of eight board-certified radiologists scoring 110 pancreatic cancers, interobserver agreement on resectability was only moderate (Fleiss κ 0.48; 95% CI 0.45–0.50), all eight readers agreed in just 30% of cases, and the margin-negative (R0) resection rate among tumors called borderline resectable ranged from 0% to 74% depending on the reader (28). On the second, where readers do agree the category itself can misclassify: in a six-reader study, interreader agreement on the National Comprehensive Cancer Network criteria was strong (intraclass correlation coefficient 0.82), yet the proportion of tumors classified correctly against a consensus reference standard was only 53%–83% (29). A criterion that is read inconsistently and, where read consistently, is correct little more than half the time is not a sound basis for the pivotal treatment decision. Compounding both, cross-sectional imaging is insensitive to the small-volume peritoneal and hepatic metastases that most often account for a tumor being systemic, so a proportion of patients judged resectable on CT are found to have occult metastatic disease at staging laparoscopy or at attempted resection (30). The imaging that defines resectability is thus limited both in what it measures—vessel contact—and in what it misses—the disseminated disease that determines outcome.

### The criteria do not account for the state of the vessels

Imaging discriminates vascular involvement poorly against a variable anatomical substrate. Published accuracy is limited: in one single-center series, computed tomography detected vascular invasion with a sensitivity of only about 39% (specificity ~90%) and endoscopic ultrasound about 55% (31)—and although more recent multidetector series report higher sensitivity, the finding that triggers a borderline or unresectable classification is still frequently discordant with operative pathology. Conversely, the degree-of-contact rule maps imperfectly onto biology: in a large surgical series, a substantial minority of tumors with a >180° venous interface (28.6%) or apparent occlusion (20%) lacked histologic vein invasion at operation (32), so the radiographic sign and the pathological reality diverge in both directions. Against this, it is worth stating what a diagnostic and interventional radiologist observes but staging reports rarely remark upon: the superior mesenteric and portal veins are not uniform cylinders. They vary in caliber and contour between individuals, contour irregularity is common in disease-free vessels, and these veins are frequently narrowed or thrombosed by the tumor and its desmoplastic reaction—yet such vessels are routinely resected and reconstructed anyway. The criterion is applied as though a normative venous anatomy existed against which invasion could be scored, when the substrate is variable and the imaging insensitive.

### The measurement is also stale and operator-dependent

Two further, more practical variables—one temporal, one institutional—degrade the anatomic decision independently of any biological argument, and both were raised in review. First, the resectability judgment decays with the time between imaging and surgery. In a study of the imaging-to-surgery interval, multidetector CT correctly predicted the absence of metastatic disease in 89.3% of patients operated within 25 days but only 77.0% of those operated later, and roughly one in ten patients harbored metastases despite a recent negative scan (33). In a second cohort, no patient explored within 22 days of imaging was found unresectable, and in a separate threshold analysis the unresectability rate rose to 26.2% beyond 32 days (hazard ratio 0.42, *P* = .021) (34). The anatomic criterion is therefore applied not only to an inconsistently read and imperfectly accurate measurement but frequently to a stale one, and the discrepancy between imaging and operative findings is partly a function of delay rather than of tumor biology. Second, both the assessment of resectability and the conduct of the operation vary with local expertise, imaging protocol, and center volume; the same tumor may be called resectable at one center and unresectable at another, and outcomes after complex resection improve with volume. These are reasons to standardize and to shorten the imaging-to-surgery interval, but they are also evidence that the anatomic criterion is not the stable, reproducible boundary the algorithm treats it as. Notably, the remedy is not simply to operate on more patients to exclude false negatives—that raises the number of futile operations described below—but to add a biological read-out that does not decay with time or vary with the observer.

### The criteria are overridden in practice

The gap between the guideline paradigm and real-world surgery is itself evidence of the criteria’s limits. Venous resection and reconstruction—performed precisely when the vein is involved, that is when the anatomic criterion for straightforward resectability is not met—is now routine, and series report survival after venous resection broadly comparable to resection without venous involvement (6, 7). Two readings are possible, and both weaken the criterion. If survival is equivalent, then venous contact was not the biologically meaningful boundary the classification treats it as. If it is not, then patients are undergoing a larger, higher-risk operation on the strength of a boundary that does not hold. Either way, the anatomic line that governs the treatment algorithm is not the line that governs outcome.

## Failure point II—occult metastasis and the limits of local cure

The second and most consequential failure is that surgery, the modality around which the paradigm is built, treats a local target in a disease whose lethality is systemic. This is not an argument that surgery never helps—it cures a genuine minority whose disease is truly confined—but that the paradigm applies it to many patients in whom the disease is not confined, and identifies that minority only in retrospect.

### “Localized” disease is usually systemic

The clearest evidence is the fate of patients after a technically complete (R0) resection of node-negative, apparently localized disease. Recurrence is the norm rather than the exception, and it is predominantly distant and early: in a large single-institution series, 76.7% of resected patients recurred at a median recurrence-free survival of 11.7 months, with distant sites dominating (35, 36). Disease that recurs distantly within months of a complete local resection was, by definition, systemic at the time of operation. The point holds even at the earliest detectable stage. Among stage IA tumors—the smallest, most favorable category—10-year overall survival is only about 49% (for patients diagnosed in 2007), even as 5-year survival has risen to ~84% in a more recent diagnosis cohort (2012); the two figures come from different diagnosis-year cohorts and are not a single within-cohort trajectory (37). That roughly half of patients with the smallest detectable pancreatic cancers are dead by 10 years indicates that occult dissemination is common even when the tumor is caught as early as current imaging allows. A further route of spread that anatomic staging does not measure is perineural invasion: in a multicenter series of 778 resected pancreatic head adenocarcinomas, perineural invasion was present in 87% of tumors overall and in 70.6% even of margin-negative, node-negative (R0/N0) tumors, where it was the only independent predictor of disease-free survival (hazard ratio 2.2) (38). Because perineural spread tracks along nerves beyond the resection field and is mechanistically linked to epithelial-to-mesenchymal transition (39), its high prevalence in apparently early, margin-negative disease is one more reason a radiographically localized tumor is frequently not biologically local.

Molecular evidence converts this inference into direct observation. Circulating tumor DNA (ctDNA) detectable after resection—molecular residual disease (MRD)—identifies patients destined to recur and is strongly, independently prognostic, confirming that tumor persists systemically despite a macroscopically complete operation and negative conventional staging (40). Detectable postoperative circulating tumor DNA is, in effect, a laboratory readout of the occult metastatic burden the operation did not reach. The staging system that governs the treatment decision is therefore miscalibrated in the direction that matters most: it labels as local a disease that molecular assessment shows to be systemic, and does so precisely for the patients most likely to be offered an operation.

### What “cure” means here, and how it should be read

Two clarifications are needed so that the cure statistics above are not over-read, both prompted by review. First, the statistical cure fraction is defined by the return of a resected cohort’s mortality to that of an age- and sex-matched general population—a comparator that has not undergone major pancreatic resection. That comparison is standard in cure-model epidemiology, but it is not a like-for-like control, and the direction of the resulting bias is not obvious. Operated patients carry perioperative and long-term surgical mortality that the general population does not, which makes a return to population-level mortality a demanding threshold, while other features of the cohort could push the estimate the other way. The cure fraction is therefore best read as an uncertain population-level estimate rather than an individual guarantee, and it is estimate-dependent: reported values for resected patients range from 20.4% (disease-free-survival cure model) to 25.2% (SEER) (18, 19). Second, cure and long-term survival are not the same, and the hard recurrence numbers should be stated alongside the cure fraction. In an actual 5-year analysis of upfront-resection patients, 27% were alive at 5 years but only 11% were both alive and recurrence-free (41); conversely, prognosis improves markedly for those who survive without recurrence—conditional survival analyses show the probability of surviving a further period rising substantially with each disease-free year after resection (42). None of this argues against operating on the confined minority; it argues that “resectable” and “curable” are not synonyms, and that the recurrence and conditional-survival data make that gap explicit rather than hiding it behind a single cure figure.

### The value of a local modality is bounded by whether the disease is local

When the disease is local, complete removal can cure; when it is not, complete local removal does not, because it does not address the compartment that determines survival. This is the same limitation that constrains radiotherapy, developed below: local control that does not convert into systemic control does not extend life. Surgery differs from radiotherapy in that, for the truly confined minority, its local control is complete enough to be curative—but for the systemic majority it shares radiotherapy’s limitation while adding far greater morbidity. The benefiting group is small: only ~10%–20% of patients are ever eligible for resection, and even in trial-selected patients on the most effective modern adjuvant regimen (mFOLFIRINOX in PRODIGE-24) 5-year overall survival was 43.2% versus 31.4% with gemcitabine (9), with unselected cohorts faring worse. Combining a small resectable fraction with long-term survival in a minority of those, the operation delivers durable benefit to a low-single-digit-to-low-double-digit percentage of all patients—and a large share of operations do not, in retrospect, alter the patient’s course.

### The cost of the operation, quantified

Because the operation benefits few, its harms weigh heavily in the balance. Pancreaticoduodenectomy carries roughly 30%–50% major morbidity and ~3%–5% perioperative mortality even at high-volume centers, with higher 90-day mortality reported outside them (43); long-term exocrine and endocrine insufficiency and chronic digestive morbidity are common among survivors (44). The financial cost is substantial and rises sharply with complications (**Table 2**) (45, 46). Expressed in 2026 dollars using the US medical-care Consumer Price Index (47), the median index-admission cost is approximately $32,700 and a grade-C pancreatic fistula approaches $141,000.

**Table 2:** Hospital cost of pancreaticoduodenectomy and its escalation with complications (single-institution series, 997 patients; index-admission USD).

Scenario	Reported cost, 2010–2017 (USD)	2026-equivalent (× 1.378)
No major complication	$21,306	$29,400
Median, all patients	$23,704 (range $10,988–$528,531)	$32,700
Any major complication	$40,005	$55,100
Postoperative pancreatic fistula — grade A	$32,164	$44,300
Postoperative pancreatic fistula — grade B	$50,264	$69,300
Postoperative pancreatic fistula — grade C	$102,013	$140,600

Reported figures are for operations performed 2010–2017; 2026-equivalent values are computed with the US medical-care Consumer Price Index (CPIMEDSL: 429.989 at January 2014, the study-period midpoint, to 592.554 at February 2026; factor 1.378). Cost and complication data from (45, 46); inflation index per (47).

Combined with the cure arithmetic, these costs yield an order-of-magnitude figure that captures the paradigm’s inefficiency: the cost per actual cure (**Table 3**). The median index admission is about $32,700 in 2026 dollars; divided by the resected cure fraction (20.4%–25.2%), that is roughly $130,000–$160,000 per cure for the hospital stay alone. A full care episode is larger. Taking the episode as two to three times the index admission—a stipulation rather than a measurement, but a conservative one, since inpatient care is about 64% of pancreatic-cancer spending in two independent datasets (48, 49)—and dividing again by the cure fraction gives roughly $260,000–$480,000 per cure, with a mid-case near $360,000 (18, 46). These figures are an illustration, not a formal cost-effectiveness analysis, and each is derived from a stated input by the stated arithmetic so that a reader can check it. At the system level the picture is the same. The United States spent about $2.55 billion on pancreatic cancer care in 2016 (49), and on the order of $4.2 billion in 2026 after medical care inflation and a rising case count—about $3.6 billion if the observed 1.4%-per-year decline in per-patient spending is carried forward (47, 50). Most of that is spent on care that does not result in cure. The purpose is not to attribute dollars to individual patients but to expose a ratio: billions spent, overwhelmingly to purchase added months rather than cures.

**Table 3:** The economics of current pancreatic-cancer care in the United States (illustrative; see the accompanying note on method).

Measure	Estimate
US national spend on pancreatic cancer	$2.55B in 2016 (95% CI $2.23–2.82B) → ~$4.2B in 2026 (~$3.6B if the 1.4%/yr per-patient decline is carried forward)
New cases/deaths per year (US, 2026)	67,530/52,740
Statistical cure fraction (all patients)	~5.7%
Statistical cure fraction (resected)	20.4% (DFS-based, multi-institutional) to 25.2% (SEER)
Share of national spend on care not resulting in cure	The great majority (most patients are never cured)
Cost per operation (index hospitalization, 2026)	~$32,700
Cost per actual cure (hospital stay → full episode)	~$130,000–$160,000 → ~$260,000–$480,000 (mid-case ~$360,000)
National spend ÷ pancreatic-cancer deaths (a ratio, not a per-patient cost)	~$68,000–$80,000

Figures combine national expenditure and incidence/mortality with the cure and cost estimates developed here. Sources: national expenditure (49), incidence and mortality (50), statistical cure fractions (18, 19), phase-of-care cost structure and inpatient share (48, 51), operative cost (46), inflation index (47).

### Surgery in guidelines versus surgery in reality

The paradigm’s internal tension sharpens when its two versions are compared. The guideline version restricts surgery to anatomically resectable tumors and frames it as potentially curative. The practiced version extends surgery well beyond that boundary—to borderline tumors after induction, to tumors requiring venous or arterial resection, and to irregular or thrombosed vessels—on the reasoning that aggressive local therapy offers the only chance of cure (6, 7). But this imports the confined-disease logic into precisely the patients least likely to have confined disease, because the more a tumor requires extending the operation, the more advanced and likely systemic it tends to be. The expansion of surgery is thus concentrated where its central premise is weakest.

### The neoadjuvant experiment already points toward the correction

The widespread shift toward neoadjuvant therapy is, in effect, an unplanned experiment that undercuts the immediate-surgery paradigm from within. When systemic therapy is given first, a subset of patients progresses during that interval and is thereby spared an operation that would not have helped (52); the interval functions as a biological filter, revealing systemic disease that anatomic staging did not. Importantly, the randomized data caution against treating neoadjuvant intensification as a survival strategy in its own right—NORPACT-1 and PREOPANC-2 found no survival advantage to intensified neoadjuvant regimens in (borderline) resectable disease (14, 15)—so the interval’s value is as a selection instrument, not an additional cytotoxic lever. The corrective is to make explicit what neoadjuvant practice already does implicitly: use a defined systemic interval as a prospective test of disease behavior, and let the result, not the baseline vessel anatomy, determine whether any local procedure is warranted and for whom.

## Failure point III—local control without a survival gain

The third failure is instructive because it isolates the paradigm’s core problem in a controlled setting. Radiotherapy is a local modality added to improve local control, and it does—yet across randomized trials it does not translate that local control into longer survival. In LAP07, adding chemoradiotherapy after induction chemotherapy for locally advanced disease improved local control but not overall survival (53). In the borderline-resectable Alliance A021501 trial, adding hypofractionated radiotherapy to induction mFOLFIRINOX did not improve outcomes and was associated with a lower R0 resection rate and shorter survival than chemotherapy alone (54); preoperative chemoradiotherapy strategies have likewise shown, at best, subgroup-limited benefit (52). The lesson generalizes to surgery: a local modality that improves local disease but does not address the systemic compartment does not extend survival. Radiation makes this visible because, unlike surgery, it has no confined-disease subgroup large enough to rescue its average effect.

A natural objection is that these trials used conventionally fractionated or moderately dose-escalated radiotherapy, and that ablative or stereotactic techniques might yet convert local control into survival. But this objection concedes the essential point while relocating it. If the limiting factor were insufficient local dose, better local control would rescue survival; the trials show that improved local control, achieved, does not. The problem is not the intensity of the local effect but its category: a purely cytotoxic local modality removes or sterilizes tissue without engaging the systemic compartment, and increasing the local dose does not change that. What distinguishes an immunogenic ablation from dose-escalated radiation is not that it kills the local tumor more completely but that it uses the local kill to prime systemic immunity—a difference in kind, not degree. The radiation experience is thus best read not as an argument for a better beam but as a demonstration that local control is not, by itself, the currency of survival in this disease, and that a local modality earns its place when it also does something systemic.

## Failure point IV—chemotherapy chosen without reference to a validated prognostic axis

The fourth failure is that the systemic backbone, which every patient receives, is selected almost without reference to the tumor’s biology. Multi-agent regimens—FOLFIRINOX and gemcitabine/nab-paclitaxel, and more recently NALIRIFOX—are chosen by performance status and clinician preference rather than by molecular features (8, 10). A reproducible, prognostically validated classical/basal-like distinction, measurable by GATA6, exists and is not used to inform care beyond BRCA/PALB2 and mismatch-repair status (21, 22).

Precision is required here, because the strongest form of this criticism has now been tested and not upheld. The retrospective expectation was that subtype would predict which regimen a patient should receive—that classical tumors would benefit preferentially from FOLFIRINOX, and basal-like tumors would not. The randomized PASS-01 trial evaluated this prospectively and did not confirm it. Overall survival favored gemcitabine/nab-paclitaxel (9.7 vs. 8.5 months; hazard ratio 1.57, 95% CI 1.08–2.28, *P* = .017); no subtype identified a subgroup benefiting from FOLFIRINOX; and the classical-subtype comparison favoring gemcitabine/nab-paclitaxel (13.9 vs. 9.7 months, *P* = .047) was an exploratory subgroup analysis rather than a formal test of interaction (16). The honest conclusion is therefore not that current practice ignores a proven regimen-selection tool, but something more limited and still consequential: it ignores a validated prognostic axis that should inform risk stratification, trial design, and the intensity and sequencing of care, and it forgoes the information that would identify chemoresistant biology early. A patient whose tumor is biologically likely to resist first-line therapy loses not only the months of that treatment but the window in which a more aggressive, multimodal strategy might have been deployed while performance status and disease burden were still favorable. Identifying chemoresistant biology up front is a plausible signal to consider earlier escalation rather than waiting until first-line failure has narrowed the options; this is a hypothesis (tier 3), however, not a validated strategy, since no trial has yet shown that escalating on the basis of subtype improves outcomes. That the single universal element of care is chosen with the least attention to the tumor’s measurable biology remains a real inconsistency; the corrective is to use biology for prognosis, stratification, and sequencing, not to claim a regimen-switching benefit the randomized evidence has not shown. We note that PASS-01’s overall result is, if anything, consistent with the framework’s separate rationale for positioning gemcitabine to deplete myeloid-derived suppressor cells, developed below.

## Failure point V—sequential, not integrated; and no path for the majority

The fifth failure is structural. Current care is organized as a sequence—chemotherapy, then surgery, then adjuvant chemotherapy—in which the systemic and immune dimensions of the disease are deferred behind a local procedure, and the sequence is fragile. A substantial fraction of resected patients never receive the intended adjuvant therapy (55), forfeiting the systemic treatment while retaining the operation’s harm. The cold, excluding microenvironment that makes the disease immunologically inert is not addressed at any point (26). And for the roughly 80% of patients who are never surgical candidates, the paradigm offers chemotherapy and palliation but no modality aimed at the accessible tumor they carry, which could in principle be turned into a source of systemic immunity. The structure treats the parts of the disease in an order that leaves the largest group without a definitive option.

The deferral of immune therapy is a particularly costly instance of this failure. The neoadjuvant interval, during which an intact accessible tumor is present and the patient has not yet been depleted by surgery, is the moment at which *in-situ* immunization and tumor-antigen priming would be most feasible—the tumor is available as an antigen source and the immune system is least compromised. Current sequencing tends to squander that window: it either proceeds to an operation that removes the antigen source and imposes a recovery period incompatible with immune therapy, or, in the non-surgical majority, never mobilizes the immune compartment at all. A structure that front-loaded immune conversion while the tumor was still present would use the disease’s one accessible asset—the primary lesion—at the time it is most usable.

## Synthesis—one root beneath the five failures

The five failure points are surface expressions of a single underlying error: a systemic, multi-compartment, molecularly convergent disease is treated as a local, single-compartment, molecularly anonymous one. Resectability is scored anatomically, surgery is offered as cure, radiation is added to tighten local control, chemotherapy is undifferentiated, and care is sequenced local-first—each because the local lesion is treated as the disease rather than as one manifestation of it (**Figure 3**). **Table 4** summarizes the failure points, the evidence, and the design requirement each imposes, together with the framework component that answers it.

**Figure 3:**
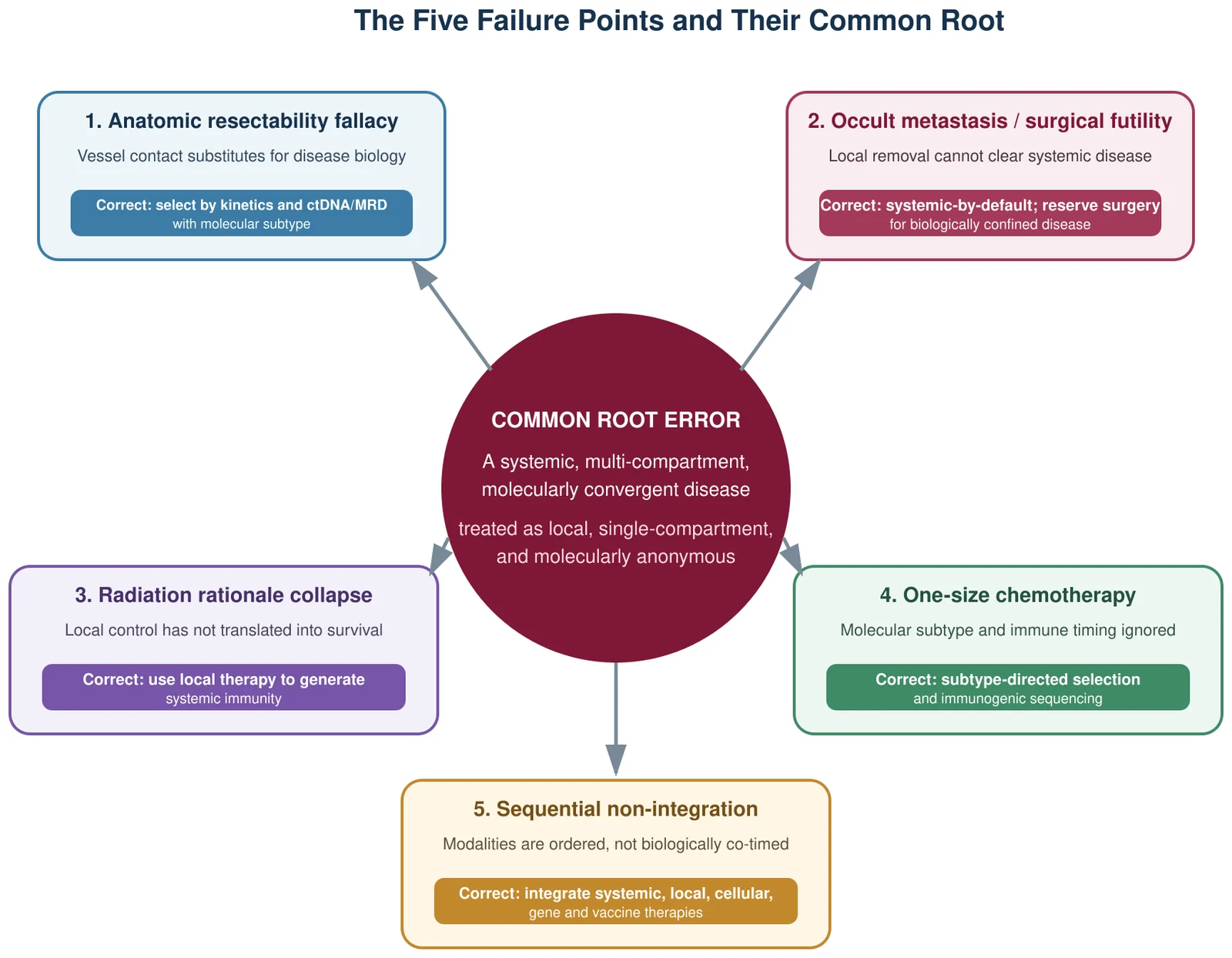
The five failure points and their common root. A concept figure arranging the five failure points (selection by anatomy, occult metastasis and the limits of local cure, local control without a survival gain, chemotherapy chosen without reference to biology, sequential non-integration) around a single central error—treating a systemic, multi-compartment, molecularly convergent disease as a local, single-compartment, anonymous one—with each failure annotated by its corrective design requirement.

**Table 4:** The five failure points of current PDAC management, their supporting evidence, the design requirement each imposes, and the framework component that answers it.

Failure point	Core evidence	What a corrected approach must do, and the framework component that answers it
I. Selection by anatomy rather than biology	Only moderate interobserver agreement (κ 0.48) and, where readers agree, correct classification in only 53%–83%; limited CT sensitivity for vascular invasion; criteria overridden by routine venous resection with comparable survival; imaging-to-surgery interval decay and volume dependence	Select by biology (response kinetics, ctDNA, subtype), not vessel contact alone → Front-end biological selection
II. Occult metastasis and the limits of local cure	Majority recur, early and distant, after R0 node-negative resection; poor 10-year survival even in stage IA; high operative morbidity/mortality/cost	Assume systemic disease by default; reserve local cure for biologically confirmed confined disease; use a low-morbidity local modality → Systemic-by-default backbone + repeatable local conversion
III. Local control without a survival gain	Added radiotherapy improves local control but not survival; worse R0 and survival in A021501	Do not pursue local control for its own sake; convert local action into systemic benefit → Ablation chosen to immunize, not to maximize local control
IV. Chemotherapy chosen without reference to biology	Validated prognostic classical/basal-like split; first-line choice ignores subtype; prospective subtype-directed selection not yet validated (PASS-01)	Use subtype for prognosis and trial stratification; escalate chemoresistant biology earlier → Subtype-informed, immunogenically sequenced systemic therapy
V. Sequential rather than integrated care; no path for the majority	Adjuvant frequently lost to surgical morbidity; cold TME untouched; no definitive option for non-surgical majority	Integrate and front-load systemic/immune therapy; provide a locoregional-immune option for unresectable disease → Integrated program + repeatable local conversion

Supporting evidence is developed and cited in the corresponding sections.

## What is needed—design principles for a corrected approach

The synthesis yields a short list of requirements that follow from the failures rather than from any particular technology. First, patient selection for local therapy should be biological and prospective, made before committing an invasive local procedure, so that the confined minority who benefit from local cure are identified in advance rather than after the fact. Second, the disease should be treated as systemic by default, with systemic and immune therapy integrated and front-loaded rather than deferred behind a local step. Third, the tumor’s convergent driver lesions should be addressed rather than left untouched, matched to the type of lesion each represents. Fourth, the local modality should be chosen not to maximize local control but to convert an accessible tumor into systemic immunity, at a fraction of the morbidity of resection. Fifth, therapy should be multi-compartment by construction, because each barrier—genetic, stromal, immune, systemic—requires its own answer, delivered in a sequence timed to their biology. The framework below is one concrete instantiation of these principles, offered as a hypothesis rather than a protocol.

### Grading the evidence

Because the framework combines elements of very different maturity, we grade each on an explicit four-tier scale before describing it, and we hold the prose to the mood each tier permits so that a randomized result and a preclinical hypothesis are not read as equivalent (**Table 5**). The four tiers are validated (tier 1, randomized support, called effective), investigational (tier 2, phase I–II or a strong signal, called promising), preclinical/mechanistic (tier 3, hypothesis-generating), and author experience (tier 4, uncontrolled single-operator experience supporting procedural feasibility only). Full definitions and the mood each licenses are in **Table 5**, and the labels propagate through the framework description, the component evidence table, and the figure legends.

**Table 5:** The evidence-tiering scheme used throughout the framework.

Tier	Label	Definition and how it constrains claims
T1	Validated	Supported by phase III or equivalent randomized evidence in PDAC or a directly analogous setting. May be described as effective.
T2	Investigational	Phase I–II proof of principle or strong mechanistic/clinical signal, not yet confirmed in randomized testing. Described as promising, not established.
T3	Preclinical/mechanistic	Rationale from *in vitro*, animal, or analogous-disease data only. Described as hypothesis-generating.
T4	Author experience	Uncontrolled, unaudited single-operator clinical experience. Supports procedural feasibility only; no efficacy or formal safety inference.

Each component below is assigned to one or more tiers, and claims about it are held to the mood the tier permits.

### A proposed multimodal, biology-guided framework

We outline the framework at the level of concept and rationale. Implementation details—vector serotypes, regulatory elements, cell-product specifications, and sequences—are deliberately omitted; the purpose is to show what each component is for and why it belongs, not to disclose a construction. The framework has four components and a selection front end, integrated in time (**Figure 4**), and the evidence tier of each is stated in **Table 6** and again at first mention, so that the reader can weigh each element accordingly.

**Figure 4:**
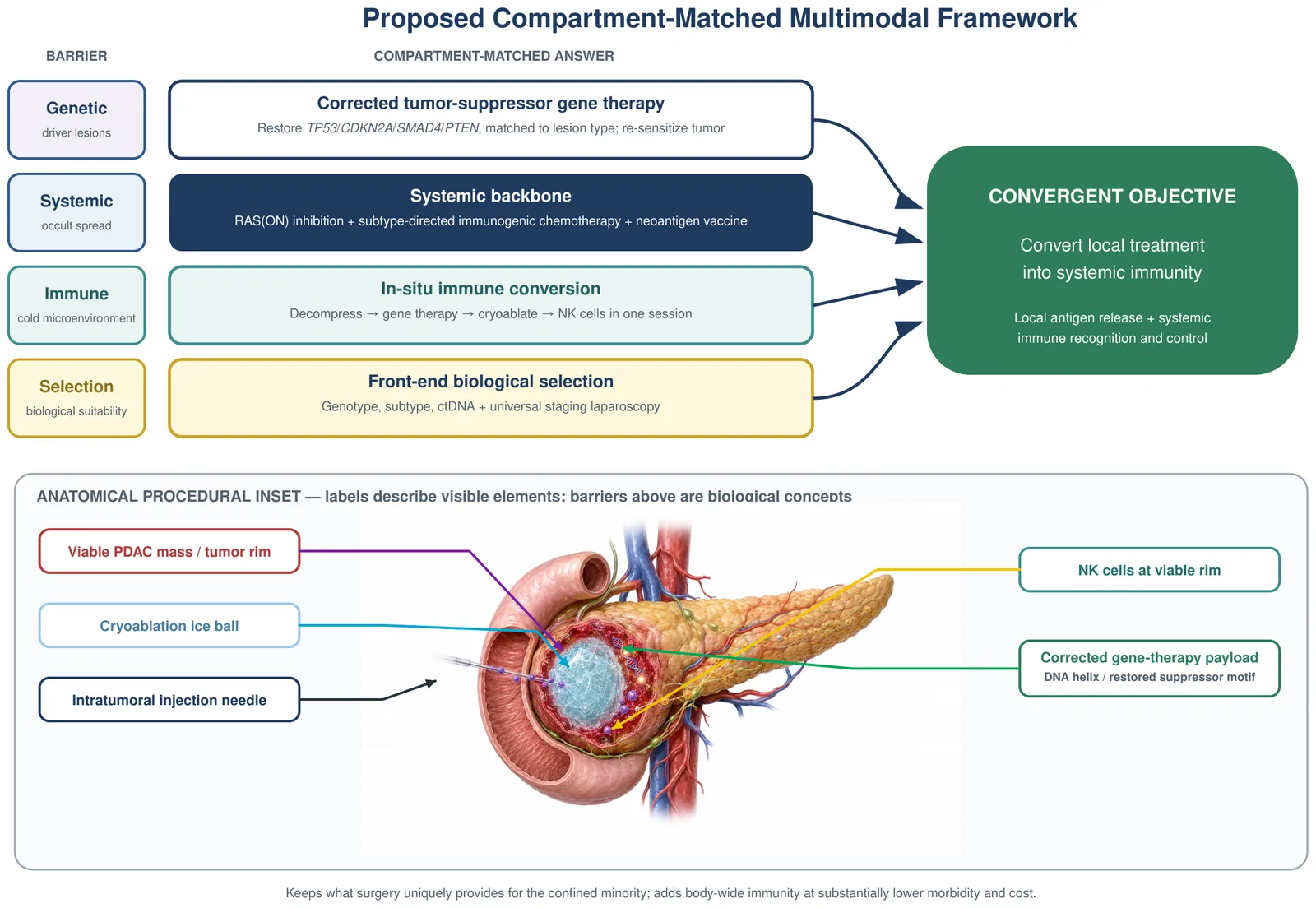
The proposed multimodal framework. The upper panel pairs each of the disease’s four barriers — genetic (driver lesions), systemic (occult spread), immune (cold microenvironment), and selection (biological suitability) — with the component that answers it: corrected tumor-suppressor gene therapy matched to lesion type (tier 3); a systemic backbone of RAS(ON) inhibition (tier 1–2), immunogenically sequenced chemotherapy (tier 1/3), and neoantigen vaccine (tier 2); cryoablation-based *in-situ* immune conversion with intratumoral priming and NK-cell delivery (tier 2/4); and front-end biological selection (tier 2). All four converge on a single objective — converting destruction of the local tumor into durable systemic immunity. The lower anatomical inset is a labeled cross-section of the accessible pancreatic tumor showing the physically visible elements of the procedure; the barrier categories in the upper panel are biological concepts and are deliberately not mapped onto the anatomy.

**Table 6:** Evidence maturity of each framework component, graded on the four-tier scheme of **Table 5**.

Component	Tier	Evidence status and basis
Front-end biological selection (ctDNA/MRD, carbohydrate antigen 19-9 (CA19-9), subtype, response kinetics)	T2	Biomarkers independently predict outcome; prospective use to assign local therapy is a selection hypothesis
RAS(ON) inhibition (daraxonrasib) backbone	T1/T2	T1 in previously treated metastatic PDAC; T2 in earlier-line, curative-intent, and immunotherapy-sequenced use
Immunogenic chemotherapy sequencing	T1/T3	Approved agents (T1); subtype-informed and immunogenic sequencing in this configuration is mechanistic (T3), and PASS-01 did not validate prospective subtype-directed regimen choice
Personalized neoantigen vaccine	T2	Phase I immunogenicity/delayed-recurrence signal; randomized adjuvant trial ongoing
Intratumoral hyaluronidase decompression	T3	Mechanistic/preclinical; local-enzyme safety by analogy; untested in this immune-conversion sequence
Cryoablation-based *in-situ* conversion + peritumoral NK cells	T2/T4	Ablation-plus-cellular-immunotherapy has a retrospective clinical signal (T2); the specific single-session sequence rests on uncontrolled author experience (T4); efficacy not established
Corrected knockdown-and-replace tumor-suppressor gene therapy	T3	Preclinical design hypothesis; not yet administered to any patient. p53-pathway restoration is biologically active in PDAC, but by wild-type addition; the knockdown-and-replace construct is unpublished
Integrated multimodal sequence (program as a whole)	T3	No direct evidence; an untested combinatorial hypothesis to be built and tested component by component before any survival claim

The grades are deliberately heterogeneous: the framework combines validated, investigational, preclinical, and author-experience elements, and the table makes that explicit.

### Corrected tumor-suppressor gene therapy, matched to lesion type (tier 3)

Because PDAC converges on a small set of inactivated suppressors, restoring them could address most tumors—but only if the restoration is designed around how each gene was lost. We propose a lesion-type rule. Genes inactivated by dominant-negative missense mutation, the paradigm being TP53, cannot be corrected by simply adding a wild-type copy, because the mutant protein poisons the restored protein and, in tumors that suppress p53 through its negative regulators, the delivered protein meets the same brake that silences the endogenous one (56). These require a knockdown-and-replace strategy: simultaneous suppression of the endogenous mutant and delivery of a regulation-resistant, suppression-immune replacement. Genes inactivated by deletion or loss of function, such as CDKN2A, SMAD4, and phosphatase and tensin homolog (PTEN), require only restoration of a functional copy. Delivery is intratumoral and tumor-restricted, and the corrected suppressors are chosen a la carte by the tumor’s genotype as determined at workup. The design intent is not durable transduction of every cell—which is not achievable and, for an immunogenic strategy, not required—but restoration of suppressor function in enough of the accessible tumor to contribute to killing and to immune visibility. This component is graded tier 3: the construct is a preclinical design hypothesis that has not been administered to any patient, and the published support establishes only that p53-pathway restoration is biologically active in PDAC, by wild-type addition, in cell lines and xenografts (57, 58).

Three features distinguish this design from the three decades of p53 gene-replacement attempts that underperformed. First, the correction is engineered to survive the environment it enters: a replacement suppressor that is degraded, dominantly poisoned, or transcriptionally silenced by the same machinery that disabled the endogenous gene will fail exactly as naive replacement has (56); the design therefore prioritizes regulation-resistance and resistance to dominant-negative interference over mere delivery of a wild-type sequence. Second, several convergent lesions are functionally coupled, so that correcting one reinforces another—restoring the CDKN2A locus re-imposes cell-cycle control and, through its alternate reading frame [the p14 alternate reading frame product, which inhibits mouse double minute 2 (MDM2)], additionally stabilizes p53, so a single locus correction acts on two axes at once.

A third feature is arguably the most consequential for the rest of the program: restoring suppressor function is hypothesized not only to kill transduced cells but to re-sensitize the tumor to therapies it previously resisted. Much of PDAC’s resistance to chemotherapy and radiation is a downstream consequence of a disabled apoptotic program—a cell that cannot execute p53-dependent death survives genotoxic insult regardless of dose. Reinstating a functional, regulation-resistant p53 is expected to restore that apoptotic competence, so that chemotherapy and radiation that were previously ineffective could become effective at lower, better-tolerated doses; a modified p53 that resists degradation has been shown to convert chemoresistant tumor cells into chemosensitive ones and to synergize with doxorubicin and cisplatin in model systems (56). Restoring suppressor function and releasing tumor antigen may also raise immunogenicity in a tumor that is otherwise immunologically inert (59). Correction is therefore intended as a conditioning step that widens the therapeutic window of the whole program rather than as a stand-alone modality, which is why it is positioned before the biologically driven chemotherapy and immunotherapy. This rationale is mechanistic (tier 3) and is to be tested, not assumed.

### A systemic backbone that treats the disease as systemic (tiers 1–3)

Because the disease is systemic by default, a systemic backbone carries the plan. It has three elements. The first is pharmacologic inhibition of the RAS pathway, now validated in previously treated metastatic disease (tier 1), with its role in earlier-line, curative-intent, and immunotherapy-sequenced settings remaining investigational (tier 2). In RASolute 302, the RAS(ON) multi-selective inhibitor daraxonrasib approximately doubled median overall survival versus chemotherapy (13.2 vs. 6.7 months; hazard ratio 0.40) (11), and oncogenic-KRAS inhibition additionally reprograms the microenvironment toward a more immune-permissive state in models (60). The second is chemotherapy sequenced for immunological effect as well as cytotoxicity: an oxaliplatin-containing regimen front-loaded to exploit immunogenic cell death (59), and gemcitabine positioned to deplete myeloid-derived suppressor cells and open a window for effector therapy (61). Because PASS-01 did not validate prospective subtype-directed regimen choice (16), the backbone uses subtype for prognosis and stratification and sequences approved agents for immune effect rather than claiming a subtype-matched regimen advantage. The third is personalized neoantigen vaccination, for which PDAC has phase I proof of principle (tier 2): individualized mRNA neoantigen vaccines induce durable T-cell responses that correlate with delayed recurrence after resection (12, 13). The leading exemplar (autogene cevumeran) is a uridine mRNA-lipoplex encoding up to 20 tumor-specific neoantigens per patient, built from whole-exome and RNA sequencing and human leukocyte antigen (HLA) typing. It has a real lead time: in the adjuvant pancreatic trial the median time from surgery to first vaccine dose was about 9.4 weeks, against an *a priori* manufacturing benchmark of 6 weeks or less, and the manufacturing input was the resection specimen (12). This lead time is why the workup that starts it must be the first step of the program. Whether a percutaneous core biopsy can supply sufficient tumor content on a comparable schedule is an assumption the first-in-human program must test. The vaccine’s per-course cost is not established; the only approved therapeutic cancer vaccine (sipuleucel-T, an autologous cellular product in a different disease) launched at roughly $93,000 per course in 2010, which is an imperfect reference point—a different modality, unadjusted for inflation—for an individualized mRNA product.

The three elements are chosen to interlock rather than merely coexist. RAS pathway inhibition has shown a substantial overall-survival benefit in a randomized trial in previously treated metastatic disease (11) and is also a conditioning agent that, in models, warms the microenvironment for the immune components (60), so it is positioned continuously and early. Immunogenic-cell-death-inducing chemotherapy is front-loaded to seed antigen and prime dendritic cells before vaccination (59), and myeloid-suppressor-depleting agents are placed to open the window into which effector cells and vaccine are delivered (61). The vaccine, whose manufacture requires a lead time, is queued at enrollment so that it is available when local conversion has maximized antigen availability.

### *In-situ* immune conversion of an accessible tumor (tier 2/4)

The local modality is chosen to convert, not merely to control. An accessible tumor is first decompressed with intratumoral hyaluronidase, which degrades the hyaluronan that saturates the PDAC stroma; because this hyaluronan, with high interstitial fluid pressure, collapses intratumoral vasculature and blocks delivery, its degradation lowers pressure, re-expands perfusion, and opens the matrix so that subsequently delivered agents distribute through the tumor (24). It is important to distinguish this use from the one that failed in the clinic. Systemic pegvorhyaluronidase alfa (PEGPH20), given intravenously to degrade hyaluronan body-wide, was tested on exactly the delivery rationale invoked here. Mechanistically it worked: in HALO-202 it improved progression-free survival, more so in hyaluronan-high tumors, and raised the response rate (62); in phase III HALO-301 it again increased the response rate (47% vs. 36%) (63). Yet, overall survival was flat (11.2 vs. 11.5 months; hazard ratio 1.00) and the program was halted (63). That dissociation corroborates this paper’s general thesis: improving delivery to and cytoreduction of the accessible primary did what it was designed to do and still did not prolong life, because what kills in this disease is the micrometastatic burden a better perfused primary does not touch.

Two features of the PEGPH20 failure are artifacts of the systemic, sustained route, and both are intended to be reduced by a single intratumoral dose. First, degrading ubiquitous hyaluronan body-wide for months carries its own morbidity: HALO-202 saw musculoskeletal toxicity and an early excess of thromboembolic events severe enough to trigger a clinical hold (62). Second, chronically stripping the stroma may be counterproductive: genetic depletion of the myofibroblast/matrix compartment produces more invasive, immunosuppressed tumors and shortens survival, because the desmoplastic matrix is also a restraint on tumor cells (64). The approach here differs on every variable: a single, transient dose into one accessible lesion, designed for local, short-lived decompression with far lower total-body exposure. Several unknowns remain, however: intratumoral pharmacokinetics, systemic spillover, and hyaluronan reconstitution are unmeasured, and any matrix disruption perturbs a compartment that is protective as well as obstructive and so must be measured rather than assumed benign (64). Decisively, the enzyme is deployed here not to potentiate cytoreduction—the endpoint on which the systemic strategy already succeeded without benefit—but to distribute a gene vector and effector cells to convert the lesion into systemic immunity, the axis PEGPH20 never tested.

The hyaluronidase action is transient—hours, not days—so it is timed to the procedure, opening the tumor bed as agents are injected, and it can be re-used at each subsequent procedure. Into the decompressed tumor the corrected gene therapy is distributed immediately—prioritizing the viable periphery but also seeding the deeper mass—through a single straight intratumoral needle that is repositioned along its track so that vector is deposited at several depths within the lesion. The tumor is then ablated by a necrosis-dominant method, image-guided percutaneous cryoablation, which releases intact tumor antigen and triggers innate danger signaling, converting the lesion into an *in-situ* vaccine rather than a sterilized scar. The design assumes that freezing does not inactivate vector distributed to the viable peripheral rim, which continues to express it—an assumption the development program must test directly (below)—so gene therapy and ablation are performed in one session (**Figure 5**). Finally, NK cells are delivered at the periphery of the ice ball, which is left frozen so that its margin can be identified on imaging—the viable interface where released antigen and incoming effectors meet; this cryoablation-plus-NK combination has clinical precedent in advanced hepatocellular carcinoma, an analogous intra-abdominal tumor (65). Unlike resection, this is a percutaneous, repeatable, low-morbidity procedure applicable to the locally advanced and metastatic majority who are not surgical candidates, and its purpose is explicitly systemic. It is the modality substitution that addresses Failure Points II, III, and V at once—keeping what surgery uniquely provides for the confined minority (destruction of the accessible tumor) while adding what surgery cannot (conversion of that local action into body-wide immunity), at lower cost and harm.

**Figure 5:**
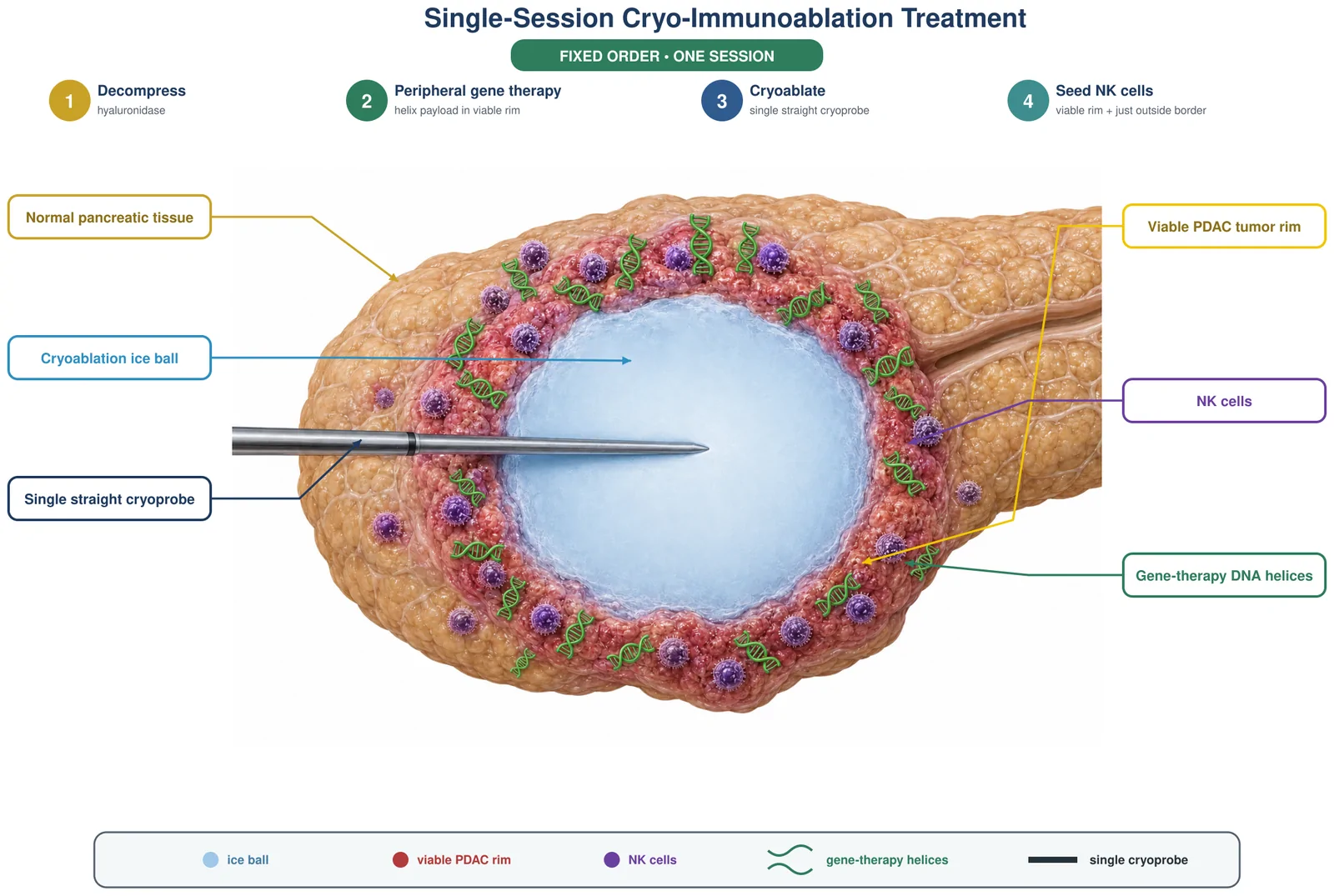
The single-session local immune-conversion procedure. Cross-sectional schematic of the fixed order of the percutaneous, image-guided session: (i) intratumoral hyaluronidase to decompress the matrix; (ii) immediate distribution of the corrected gene therapy across the lesion through a single straight intratumoral needle, repositioned along its track; (iii) cryoablation of the accessible tumor with a single straight, icepick-like cryoprobe, releasing intact antigen, with an adjacent splanchnic vessel protected by the heat-sink effect of flowing blood; and (iv) NK cells deposited at the periphery of the ice ball, which is left frozen so that its margin remains visible on imaging. The figure shows why cryoablation and gene therapy are done in one sitting and why the modality is both vessel-sparing and immunogenic.

Several practical properties make this modality suited to the disease as it presents. Because it is percutaneous and image-guided, it can be directed at tumors abutting or encasing the splanchnic vessels that make resection hazardous (**Figure 6**), and cryoablation is comparatively vessel-sparing precisely there: flowing blood in an adjacent large vessel acts as a heat sink that keeps the vessel wall from freezing to destruction. Heat-based ablation (radiofrequency, microwave) denatures antigen, and irreversible electroporation (IRE) is largely non-necrotic; both induce comparatively little antitumor immune response in preclinical and clinical series (66); trading cryoablation for them to protect a vessel would forfeit the *in-situ* vaccination that is the point, and—given the heat-sink protection—is generally unnecessary. Because its morbidity is a fraction of pancreaticoduodenectomy—percutaneous cryoablation is well established across tumor types as minimally invasive, performed on an outpatient basis or with a single overnight observation (67–69)—it does not consume the recovery interval in which systemic and immune therapy must continue, addressing the adjuvant-loss failure (55). And because it is repeatable, it can be reapplied to a new or progressing accessible lesion as on-demand re-vaccination.

**Figure 6:**
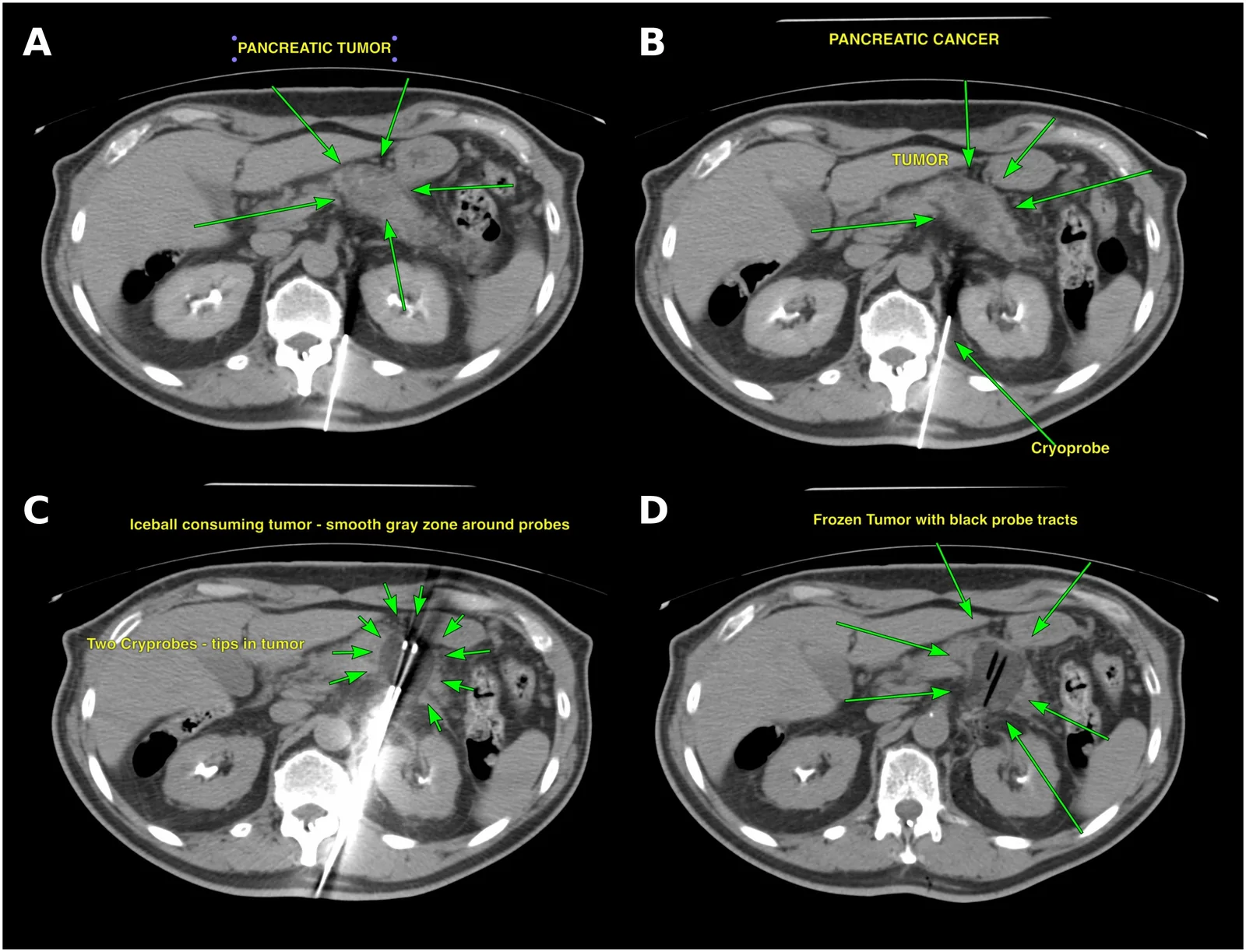
Percutaneous CT-guided cryoablation of a body-of-pancreas PDAC (representative case). Contrast-enhanced CT, delayed phase, axial images. **(A)** Baseline: a 3.5 cm biopsy-proven ductal adenocarcinoma in the body of the pancreas (arrows). **(B)** A cryoprobe advanced through a paraspinal approach between the vertebral body and the medial renal cortex. **(C)** Two cryoprobes within the tumor, surrounded by a smooth, low-density ice ball defining the ablation margin, readily monitored on CT. **(D)** After probe removal, the frozen tumor with air-filled probe tracks within the residual ice ball. The case documents that percutaneous cryoablation of a pancreatic tumor is feasible, monitored in real time, and can be directed to lesions adjacent to the great vessels and kidney—the procedural basis (tier 4) for the *in-situ* immune-conversion module (schematic, **Figure 5**). Images courtesy of the author.

The evidentiary status of the local module must be stated explicitly, because it is where the author’s own experience enters and must be labeled as such (tier 4). In its procedural elements the module is established practice: the author has performed percutaneous cryoablation of PDAC since 1999 and, in cases treated since, has combined it with intratumoral NK-cell delivery, chemotherapy, and immunotherapy, sequenced with the patient’s systemic therapy. The one element not yet deployed is the corrected adeno-associated virus (AAV) gene therapy, which has not been administered to any patient and would follow the standard preclinical and investigational-new-drug pathway before any first-in-human use. This experience is uncontrolled, unaudited, and small in number; it supports procedural feasibility only and is not offered as evidence of efficacy, and no formal safety inference can be drawn. The claim is correspondingly narrow: the local-conversion procedure is practicable and has been practiced for over two decades; whether the integrated program improves survival is the hypothesis to be tested.

### Front-end biological selection (tier 2)

The selection front end addresses Failure Point I by replacing the anatomic decision with a biological one made prospectively, with two complementary readouts. The first and faster is direct molecular profiling—genotype, transcriptional subtype, and baseline circulating tumor DNA—which returns within a few weeks and informs prognosis, the gene-therapy design, and the sequencing of systemic therapy, with regimen choice driven by actionable alterations rather than by transcriptional subtype; this is the readout the protocol acts on. The second is the response to therapy over a defined interval, which reveals aggressive systemic biology that anatomy cannot; it informs intent rather than imposing delay, because the low-morbidity module is hypothesized to benefit patients across the spectrum and need not wait behind a prolonged induction. Selection integrates CA19–9 kinetics, ctDNA and MRD (40), molecular subtype (22), and, where appropriate, staging laparoscopy. Exploratory blood-based operability signatures are also emerging—a serum microRNA panel, for example, has been reported to distinguish operable from inoperable disease—but such signatures rest on small, single-cohort analyses without external validation (in the cited study, 21 operable vs. 79 inoperable patients) and are not yet suitable to gate a treatment decision (70). The confined minority for whom local cure is genuinely appropriate are thus identified in advance, on biology, rather than discovered after an operation whose benefit was never established.

### Integration in time

The components are not a menu but a sequence, and the sequence is paced by molecular turnaround rather than by a fixed induction period. The workup and vaccine build begin first, because the personalized vaccine has the longest lead time [a manufacturing benchmark of 6 weeks or less, and a median of ~9.4 weeks from surgery to first dose in the adjuvant trial (12)] and its workup must be started at biopsy. Systemic therapy begins as soon as biopsies are collected and is refined once sequencing returns—typically two to three weeks—using actionable alterations and immunogenic sequencing rather than transcriptional subtype to choose the regimen. The local immune-conversion procedure proceeds once the gene-therapy product and effector cells are ready, and surveillance is thereafter biomarker-led and adaptive. The unifying move is to use the patient’s own accessible tumor as a training ground for systemic immunity—the conversion of local into systemic benefit that radiation and surgery, as purely local therapies, do not achieve (**Figure 7**).

**Figure 7:**
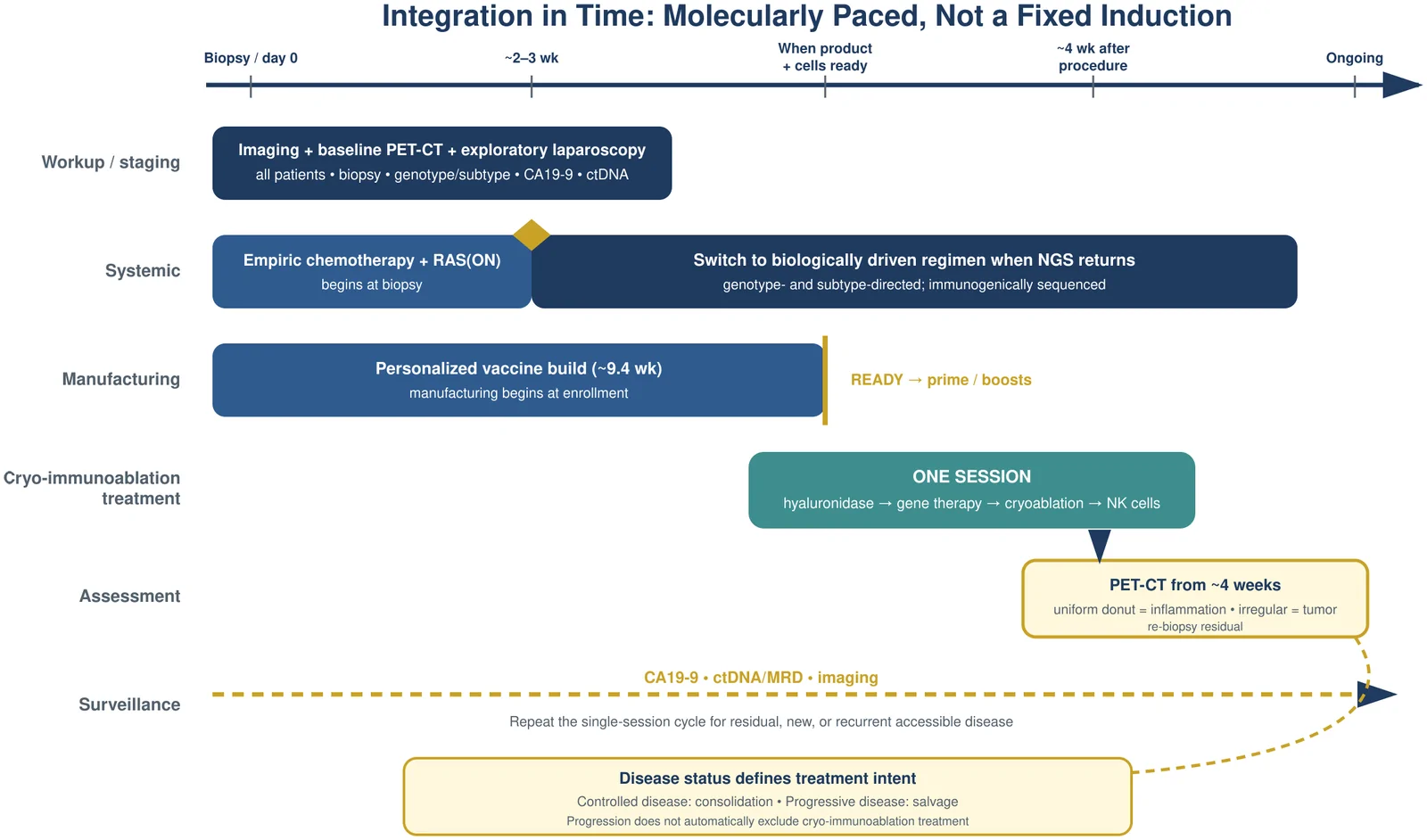
Integration in time. A timeline (relative to biopsy/day 0) showing the vaccine-build and molecular-workup lead-in, empiric systemic therapy started at biopsy and switched to a biologically driven regimen once sequencing returns (~2–3 weeks), the single-session local procedure (hyaluronidase → gene therapy → cryoablation → peritumoral NK cells), and PET-CT-based response assessment from ~4 weeks, followed by biomarker-led adaptive surveillance and repeat cycles as needed. The schedule is paced by molecular turnaround rather than a fixed induction period.

### The sequence as a staged research algorithm

The sequence below is presented deliberately as a research algorithm—a set of study steps with decision gates and off-ramps—and not as a clinical directive or a validated treatment pathway (**Figure 8**). Its purpose is to make the hypothesis concrete and testable, not to assert readiness; each step names what is done and, where relevant, the point at which a patient exits the pathway.

**Figure 8:**
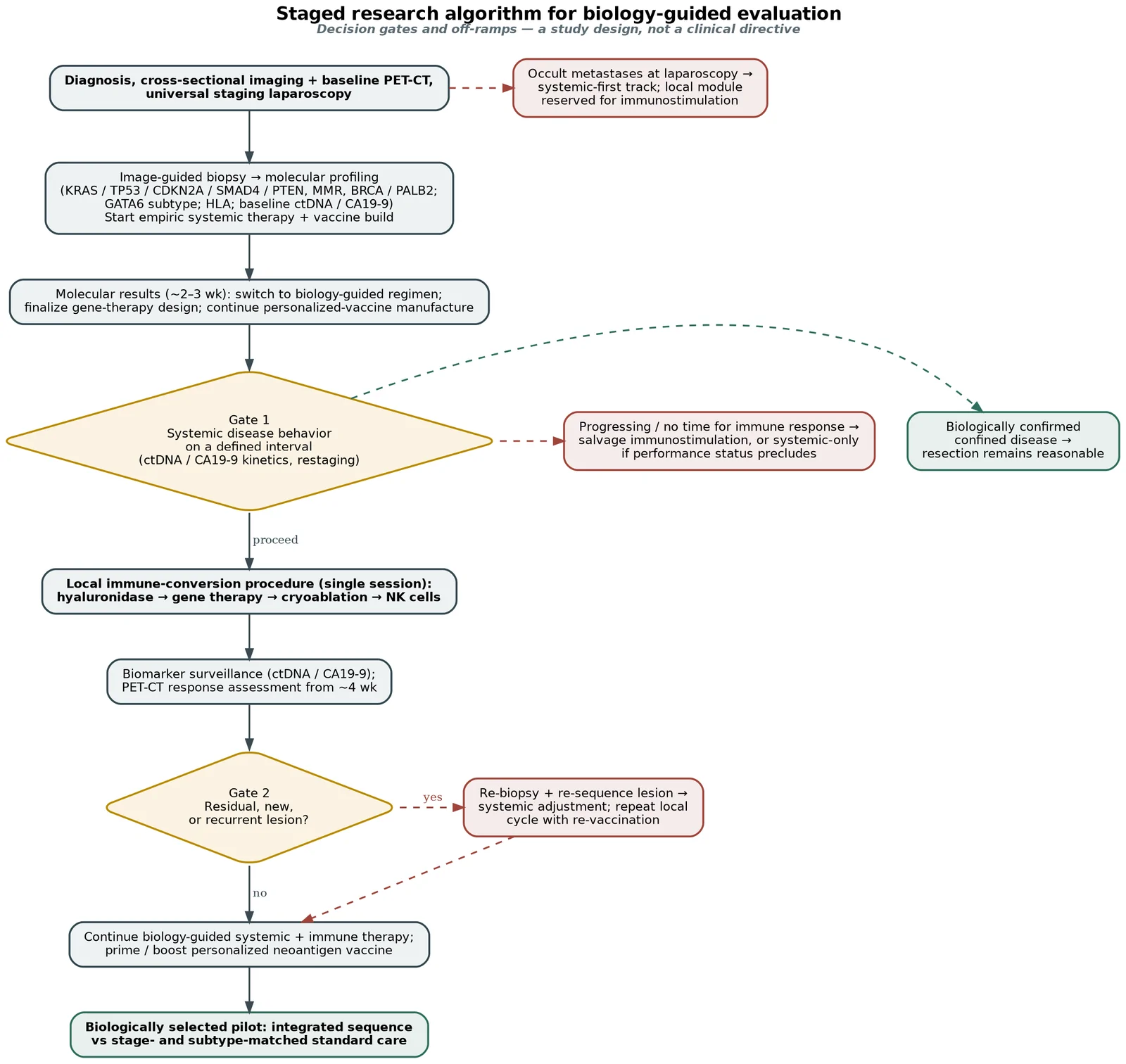
The proposed sequence as a staged research algorithm. A one-page workflow summarizing the study steps as decision gates (systemic disease behavior; residual/new lesion) and off-ramps (occult metastases at laparoscopy; progression or insufficient time for an immune response; biologically confirmed confined disease routed to resection). The figure is presented as a study design rather than a clinical directive: modules are evaluated component-wise, the first-in-human study does not combine every module in one dose-escalation, and endpoints are mechanistic and immune-correlative before any survival claim.

Two lead times govern the schedule: comprehensive tumor sequencing typically returns in about 2 to 3 weeks (up to 3 to 4 in real-world practice), while the personalized vaccine adds a manufacturing lead time—a benchmark of six weeks or less, and about 9.4 weeks from surgery to first dose in the adjuvant trial (12). Systemic therapy is begun without waiting for these results and adjusted once they return.

- Step 1—Diagnosis, cross-sectional imaging including baseline PET-CT, and staging. Exploratory (staging) laparoscopy is performed in all patients being considered for a local-cure procedure, because cross-sectional imaging misses the small-volume peritoneal and hepatic disease that most often makes the tumor systemic (30). Off-ramp: occult metastases at laparoscopy move the patient to a systemic-first track, with the local module reserved for immunostimulation rather than attempted local cure.
- Step 2—Image-guided core biopsy for molecular analysis (KRAS, TP53, CDKN2A, SMAD4, PTEN, BRCA/PALB2, mismatch-repair status; subtype by GATA6; HLA typing; baseline CA19–9 and circulating tumor DNA). Empiric systemic therapy begins as soon as biopsies are collected.
- Step 3—When molecular analysis returns (~2–3 weeks), three things happen at once: the systemic regimen is refined using the molecular profile (actionable alterations and immunogenic sequencing, not transcriptional subtype); the sequencing result defines the corrected gene-therapy design; and the personalized-vaccine build begins. There is no prolonged holding period.
- Step 4—Gate: assess systemic disease behavior on a defined interval (ctDNA/CA19–9 kinetics, restaging). Proceed to the local module when the gene-therapy product and effector cells are ready. Off-ramp: if disease is progressing rapidly or performance status leaves no time for an immune response, the intent of the module shifts to salvage immunostimulation, or the patient continues on systemic therapy alone.
- Step 5—Local procedure, single session: intratumoral hyaluronidase → gene therapy via a single straight intratumoral needle → cryoablation of all accessible tumor with a single icepick-like cryoprobe → NK cells at the periphery of the ice ball, left frozen so its margin is visible.
- Step 6—Biomarker surveillance (serial CA19–9 and ctDNA/MRD) begins after ablation.
- Step 7—Response assessment by PET-CT from ~4 weeks. A uniform thin rim of “donut” 18F-fluorodeoxyglucose (FDG) activity is expected to reflect benign post-freeze inflammation and to be distinguishable from the irregular activity of residual tumor; if residual disease is suggested, biopsy it; otherwise, reassess at ~8 weeks.
- Step 8—Continue biologically driven chemotherapy and immunotherapy and give the personalized vaccine as prime and boosts once built. Re-biopsy residual disease with repeat sequencing to confirm transduction and re-sensitization and to expose new vulnerabilities.
- Step 9—Gate: residual, new, or recurrent lesion? If yes, re-biopsy and re-sequence—a new lesion implies a clone not responding to current therapy—prompt a systemic adjustment and, where accessible, a repeat local cycle with re-vaccination against the evolved antigen profile. If no, continue biology-guided systemic and immune therapy.

Beginning systemic therapy before molecular results return carries a small cost—a few patients may receive a less-than-optimal first regimen for 2–3 weeks before adjustment—which is minor against the benefit of not losing time and is minimized by switching the moment results arrive.

## Clinical feasibility and a staged development program

A framework of this complexity must be judged not only on mechanism but on whether it can be built, regulated, manufactured, selected for, and paid for. This section addresses those barriers directly and specifies a staged first-in-human program, because the appropriate answer to “does this imply clinical readiness?” is an explicit development path that does not (**Table 7**).

**Table 7:** A staged first-in-human development program.

Module (evidence tier)	First-in-human design anchor and endpoint	Go/no-go gate
Front-end biological selection (T2)	Prospective trial assigning local therapy on biology (response kinetics, ctDNA, subtype) vs. baseline anatomy; approved modalities only; endpoint = futile-procedure rate and outcome by assignment	Proceed if biological assignment reduces futile local procedures without survival decrement
Intratumoral gene transfer (T3)	Dose-finding of the corrected construct alone, EUS/percutaneous, on the CYL-02 and CAN-2409 precedents; endpoint = safety, biodistribution, transduction	Proceed only after acceptable safety and demonstrated intratumoral expression
Cryoablation + peritumoral NK + hyaluronidase (T2/T4)	Single-session local module without the novel gene therapy; endpoint = safety and immune-correlative conversion (paired biopsy, ctDNA, T-cell response)	Proceed if systemic immune activation is measurable at acceptable toxicity
Personalized neoantigen vaccine (T2)	Window-of-opportunity add-on using the demonstrated manufacture-and-deliver interval (≤6-week benchmark; ~9.4 weeks surgery-to-dose in the adjuvant trial); endpoint = neoantigen-specific T-cell response	Proceed if functional T-cell responses are induced on schedule
Integrated sequence (T3)	Biologically selected pilot vs. stage- and subtype-matched standard care, assembled only after each module clears its gate; not a single stacked dose-escalation	Survival claim deferred until mechanism and safety are established module by module

Each module is de-risked separately against a real precedent and a defined endpoint, and advances only through an explicit go/no-go gate; the integrated sequence is assembled last and is not tested as a single stacked dose-escalation. Evidence tiers as in **Table 5**.

## Implementation barriers, stated plainly

The program combines modalities that ordinarily sit under different regulatory and manufacturing regimes: an investigational AAV gene therapy and a cell product [each a biologic requiring its own investigational new drug (IND) application and chemistry, manufacturing, and controls (CMC) package], an intratumoral enzyme, an ablation device, and a personalized mRNA vaccine with a bespoke, per-patient manufacturing chain. Each carries its own release testing, cost, and cold-chain and scheduling logistics, and the personalized vaccine’s manufacturing lead time (a 6-week-or-less benchmark, with about 9.4 weeks from surgery to first dose observed in the adjuvant trial) imposes a fixed floor on the timeline (12). Patient selection is itself a barrier and an asset: the program requires up-front molecular profiling, an accessible lesion suitable for percutaneous injection and ablation, adequate performance status, and—for the immune components—enough anticipated survival for an immune response to mature. Cost is non-trivial: the vaccine’s per-course cost is not established (the only approved therapeutic cancer vaccine, sipuleucel-T, a different modality, launched at roughly $93,000 in 2010), and it is additional to the gene therapy, cell product, and procedural costs. These barriers do not argue against testing the framework, but they do argue decisively against testing it all at once.

### A staged first-in-human program, not a single combined study

The cardinal rule of the development path is that the first integrated clinical study must not combine an untested AAV construct, a cell product, an intratumoral enzyme, an ablation, a personalized vaccine, and a targeted drug in a single dose-escalation. The number of interacting toxicities and the impossibility of attributing an adverse event would make such a study both unsafe and uninformative. The components are therefore developed and de-risked separately and assembled only after each clears a defined gate (**Table 7**).

The staged design is anchored on real precedents rather than on assumption. Intratumoral gene transfer in PDAC is feasible. The CYL-02 non-viral gene therapy was delivered by endoscopic-ultrasound-guided intratumoral injection with gemcitabine in a completed phase I trial (71). The adenoviral herpes simplex virus thymidine-kinase agent now known as CAN-2409 was given intratumorally with a prodrug as an adjunct to surgery or chemoradiation in PDAC, with acceptable safety and a measurable increase in intratumoral CD8+ T-cell infiltration (72). These establish that the gene-transfer step of the local module rests on an existing safety and delivery base, onto which the corrected construct is the novel increment to be tested alone first. The personalized-vaccine step can be evaluated in a window-of-opportunity design that exploits the demonstrated manufacture-and-deliver interval (about 9.4 weeks from surgery to first dose in the adjuvant trial) (12). The selection thesis—the least agent-encumbered and most immediately testable element—can be tested with approved modalities in a trial that assigns local therapy on biological rather than anatomic criteria.

The efficacy stages are sized as designs, not aspirations. A single-arm mechanistic study of *in-situ* conversion can be powered as a Simon two-stage design on a response-defined endpoint. For illustrative parameters (unacceptable response 0.15, target 0.40, α 0.05, power 0.80), an optimal two-stage design enrolls 7 patients in the first stage, stops for futility if ≤ 1 responds, and proceeds to 25 total, positive if ≥ 7 respond, with expected sample size ~12 under the null (73). A paired-biopsy mechanistic endpoint (pre- and post-conversion tumor and blood, testing for measurable systemic immune activation) is estimable with on the order of 14 evaluable patients. Only after each module has cleared its safety and mechanism gate is the integrated sequence tested in a biologically selected pilot against stage- and subtype-matched standard care.

### Relationship to standard of care

The framework is not a repudiation of every element of current care but a re-architecting of how the elements relate. It retains chemotherapy and adds the RAS(ON) backbone (validated in metastatic disease), sequences them for immune effect, and uses biology for prognosis and stratification—with regimen choice driven by actionable alterations rather than by transcriptional subtype. It retains a role for destroying the accessible tumor but substitutes a low-morbidity, immunogenic, repeatable modality for resection in all but the biologically confirmed confined minority, for whom surgery remains reasonable. It treats radiotherapy as a local-control measure rather than a survival strategy, on the evidence that local control alone does not extend life (53, 54). And it adds what standard care lacks: correction of the convergent driver lesions, conversion of the tumor into an immunizing event, and a definitive option under study for the unresectable majority. Each change is directional—from anatomic to biological selection, from untreated to corrected drivers, from high-morbidity resection to immunogenic ablation, and from palliation-only to a locoregional-immune program for the majority—and each element carries the evidence tier assigned in **Table 6**, not a claim of parity with validated care.

## Relationship to prior and parallel work

The organizing insight of the local module—that destroying an accessible tumor can be turned from a local maneuver into a systemic immune event—is not new, and the framework is strengthened by situating it against the work that established it. The most direct precedent is clinical. Niu, Xu, and colleagues reported percutaneous cryoablation combined with cellular immunotherapy in metastatic pancreatic cancer, finding in a retrospective, non-randomized comparison that cryoimmunotherapy was associated with longer median overall survival than cryoablation alone, immunotherapy alone, or chemotherapy, with repeated ablation and intact baseline immune function predicting better outcome (74). This body of experience has since been reviewed under the heading of cryoimmunotherapy (75). In parallel, IRE has become the most-studied ablation partner for immunotherapy in locally advanced disease, combined with checkpoint inhibitors, NK cells, and other immunostimulants on the rationale that local ablation can provoke a systemic response (66). Preclinical models show that IRE paired with an intratumoral immunostimulant can control untreated, off-target metastatic lesions (76), and an active clinical program is testing IRE with pembrolizumab under blood- and tumor-based immune-correlative endpoints (77). On the systemic-vaccine side, the personalized neoantigen-vaccine principle is itself advancing toward randomized testing in a phase II adjuvant trial in resected PDAC (78). This prior and parallel work establishes that ablation-plus-immunotherapy and personalized vaccination in pancreatic cancer are feasible and signal activity, and it sets the bar the present proposal must clear.

Against that background, the shared principle is *in-situ* immune conversion. What is new is set out in “What This Article Adds”: the deliberate choice of cryoablation over IRE or heat for antigen-preserving, vessel-sparing immunogenic ablation, the addition of corrected gene therapy into the same lesion at the same session, integration with a biologically selected and immunogenically sequenced backbone, and selection by biology rather than anatomy. Whether IRE can be rendered immunogenic by other means remains an open empirical question. The earlier work is therefore a foundation rather than a rival: it demonstrates the principle at the level of feasibility and signal, and the present framework proposes the more complete, mechanistically specified integration that the prior results invite but do not themselves provide.

## Limitations and what validation requires

The critique and the proposal stand on different evidentiary footings, and we distinguish them deliberately. The critique rests on published, largely uncontested data: the epidemiology, the recurrence patterns after resection, the randomized radiation trials, the subtype literature, and the operative morbidity and cost figures are drawn from the existing record. The interpretive claims built on them—that anatomic resectability is the wrong decision variable for most patients, that local cure is applied to many in whom the disease is not confined—are strong readings of that record. We have noted where surgeons would reasonably contest them, chiefly regarding the confined minority for whom surgery is genuinely curative and whom the framework explicitly preserves.

The proposal is a hypothesis, and it is worth naming its load-bearing assumptions and the observations that would falsify each, so that it can be tested rather than merely believed. First, it assumes that a single intratumoral dose of hyaluronidase can decompress the matrix enough to enable vector and cell distribution without the toxicity or pro-metastatic penalty seen with sustained systemic stromal depletion; this is falsified if measured intratumoral distribution is not improved, or if local matrix disruption accelerates progression as the genetic-depletion models warn (64). Second, it assumes both that vector delivered to the viable rim survives the freeze and continues to express, and that cryoablation of an accessible lesion, with local gene therapy and NK delivery, produces measurable systemic immune activation. This is falsified if post-ablation biopsy shows no transgene expression in the surviving rim, or if paired-biopsy and circulating immune endpoints show no conversion. Third, it assumes that a corrected, regulation-resistant suppressor can be delivered and expressed in enough of the tumor to condition it; this is falsified if the construct fails to express or to re-sensitize in first-in-human testing. Fourth, it assumes that biological selection reduces futile local procedures without a survival decrement relative to anatomic selection; this is falsified if a selection trial shows no reduction in futile procedures or worse outcomes. Fifth, and most globally, it assumes that assembling these parts yields systemic immunity rather than additive cost and toxicity without additive benefit—a general risk against which the PEGPH20 experience, where a well-founded delivery strategy improved every intermediate endpoint and still did not extend life, is a standing warning (63). The central testable prediction is specific and falsifiable: that biological rather than anatomic selection, combined with modality substitution that converts local treatment into systemic immunity, will extend survival where local control alone has not.

The development path follows the staging set out in the feasibility section. The selection thesis is the most immediately testable and least encumbered by novel agents and could be run largely with approved modalities. The immune-conversion thesis can be built component-wise from elements with independent safety and mechanism data, with systemic immune conversion demonstrated on immune-monitoring endpoints before any survival claim. The corrected gene therapy proceeds on the standard investigational path for a novel biologic. This staging lets the parts that are ready—above all the replacement of anatomic with biological selection—be tested and, if validated, adopted well before the more novel components mature.

## Discussion

It is worth stating plainly how this proposal should be weighed, and where a fair reader should remain unconvinced. Its principal vulnerability is its integration: it assembles several components—corrected gene therapy, a RAS(ON) backbone, immunogenic cryoablation, peritumoral NK cells, and a personalized neoantigen vaccine—none individually novel, but whose combination has not been tested and whose interactions cannot be fully predicted from the parts. Each added element multiplies the toxicity surface, the manufacturing and regulatory burden, and the number of ways the whole can fail, and oncology’s history is full of mechanistically attractive combinations that did not survive a randomized trial; the PEGPH20 experience is a standing warning against inferring survival from mechanism (63). We judge the risk worth taking—because the paradigm being replaced is itself underperforming, and because the framework is designed to be built and tested component by component rather than deployed whole—but we do not claim the outcome is known.

The argument of this paper is therefore asymmetric by design. The case that the current local paradigm underperforms rests on established, largely uncontested data, and we advance it with confidence. The case that this particular framework will succeed rests on a hypothesis, and we advance it as one—falsifiable, stageable, and to be judged by whether biological selection and immune-converting local therapy extend survival where local control alone has not. The value of the proposal does not depend on its being correct in every particular; it depends on its being tested, and on the reframing it forces even if the specific construction proves wrong: that the disease is systemic and should be treated as such.

A final caution belongs to the reader. This synthesis is the work of a single author who is the founder and chief executive of a company developing related approaches and the physician whose procedural experience is cited (see Conflict of interest). The critique is built on published data any reader can independently check, but the absence of an independent co-author to check evenhandedness is itself a limitation, and the reader should weight the conflicted, single-author character of the work accordingly. Nothing here should be read as a recommendation for clinical adoption ahead of the staged, independently conducted studies described above.

Pancreatic cancer is treated as a local disease largely because that is how its treatment grew up. The result is a paradigm whose central decision—anatomic resectability—rests on a variable that is imperfectly predictive, decays with time, varies between centers, and is routinely overridden in practice. Its defining modality cures a minority while harming many; its radiation strategy has not been shown to extend life; its chemotherapy under-uses the prognostic information its biology provides; and it leaves most patients without a definitive option. These are best understood not as independent shortcomings but as one error seen 5 times. The corrective is to select patients by biology rather than anatomy, to treat the disease as systemic by default, to address its convergent lesions, and to substitute for resection a local modality whose purpose is to convert an accessible tumor into systemic immunity. Whether the specific framework proposed here succeeds is an empirical question to be settled by the studies it specifies; that the paradigm it seeks to replace is underperforming is well supported by the evidence assembled here. The objective should shift from removing the tumor to converting local disease into systemic benefit—the step the current approach, by its structure, is not designed to take.

## Data Availability

Publicly available datasets were analyzed in this study. This data can be found here: pubmed - see references.

## References

[1] Tempero MA Malafa MP Al-Hawary M Behrman SW Benson AB Cardin DB . Pancreatic adenocarcinoma, version 2.2021, NCCN clinical practice guidelines in oncology. J Natl Compr Canc Netw. (2021) 19:439–57. doi: 10.6004/jnccn.2021.0017 33845462

[2] Bailey P Chang DK Nones K Johns AL Patch AM Gingras MC . Genomic analyses identify molecular subtypes of pancreatic cancer. Nature. (2016) 531:47–52. doi: 10.1038/nature16965 26909576

[3] Cancer Genome Atlas Research Network . Integrated genomic characterization of pancreatic ductal adenocarcinoma. Cancer Cell. (2017) 32:185–203. doi: 10.1016/j.ccell.2017.07.007 28810144 PMC5964983

[4] Öhlund D Handly-Santana A Biffi G Elyada E Almeida AS Ponz-Sarvise M . Distinct populations of inflammatory fibroblasts and myofibroblasts in pancreatic cancer. J Exp Med. (2017) 214:579–96. doi: 10.1084/jem.20162024 28232471 PMC5339682

[5] Jamal MH Khan MN . Developments in pancreatic cancer emerging therapies, diagnostic methods, and epidemiology. Pathol Res Pract. (2025) 271:156012. doi: 10.1016/j.prp.2025.156012 40414076

[6] Wang WL Ye S Yan S Shen Y Zhang M Wu J . Pancreaticoduodenectomy with portal vein/superior mesenteric vein resection for patients with pancreatic cancer with venous invasion. Hepatobiliary Pancreat Dis Int. (2015) 14:429–35. doi: 10.1016/s1499-3872(15)60400-3 26256089

[7] Mascoli C D'Ambra M Casadei R Ricci C Pezzilli R Calculli L . Portal/superior mesenteric vein reconstruction during pancreatic resection using a cryopreserved arterial homograft. Dig Surg. (2015) 32:284–90. doi: 10.1159/000381194 26113314

[8] Conroy T Hammel P Hebbar M Ben Abdelghani M Wei AC Raoul JL . FOLFIRINOX or gemcitabine as adjuvant therapy for pancreatic cancer (PRODIGE 24/CCTG PA.6). N Engl J Med. (2018) 379:2395–406. doi: 10.1056/NEJMoa1809775 30575490

[9] Conroy T Castan F Lopez A Turpin A Ben Abdelghani M Wei AC . Five-year outcomes of FOLFIRINOX vs gemcitabine as adjuvant therapy for pancreatic cancer (PRODIGE 24/CCTG PA.6): a randomized clinical trial. JAMA Oncol. (2022) 8:1571–8. doi: 10.1001/jamaoncol.2022.3829 36048453 PMC9437831

[10] Wainberg ZA Melisi D Macarulla T Pazo Cid R Chandana SR De La Fouchardière C . NALIRIFOX versus nab-paclitaxel and gemcitabine in treatment-naive patients with metastatic pancreatic ductal adenocarcinoma (NAPOLI 3): a randomised, open-label, phase 3 trial. Lancet. (2023) 402:1272–81. doi: 10.1016/S0140-6736(23)01366-1 37708904 PMC11664154

[11] O'Reilly EM Wainberg ZA Hendifar AE Borad MJ Pietrantonio F Pant S . Daraxonrasib or chemotherapy in previously treated metastatic pancreatic cancer. N Engl J Med. (2026) 395:325–37. doi: 10.1056/NEJMoa2605555 42223072

[12] Rojas LA Sethna Z Soares KC Olcese C Pang N Patterson E . Personalized RNA neoantigen vaccines stimulate T cells in pancreatic cancer. Nature. (2023) 618:144–50. doi: 10.1038/s41586-023-06063-y 37165196 PMC10171177

[13] Sethna Z Guasp P Reiche C Milighetti M Ceglia N Patterson E . RNA neoantigen vaccines prime long-lived CD8+ T cells in pancreatic cancer. Nature. (2025) 639:1042–51. doi: 10.1038/s41586-024-08508-4 39972124 PMC11946889

[14] Labori KJ Bratlie SO Andersson B Angelsen JH Biörserud C Björnsson B . Neoadjuvant FOLFIRINOX versus upfront surgery for resectable pancreatic head cancer (NORPACT-1): a multicentre, randomised, phase 2 trial. Lancet Gastroenterol Hepatol. (2024) 9:205–17. doi: 10.1016/S2468-1253(23)00405-3 38237621

[15] Janssen QP van Dam JL van Bekkum ML Bonsing BA Bos H Bosscha KP . Neoadjuvant FOLFIRINOX versus neoadjuvant gemcitabine-based chemoradiotherapy in resectable and borderline resectable pancreatic cancer (PREOPANC-2): a multicentre, open-label, phase 3 randomised trial. Lancet Oncol. (2025) 26:1346–56. doi: 10.1016/S1470-2045(25)00363-8 40945523

[16] Knox JJ O'Kane G King D Laheru D Habowski AN Yu K . PASS-01: randomized phase II trial of modified FOLFIRINOX versus gemcitabine/nab-paclitaxel and molecular correlatives for previously untreated metastatic pancreatic cancer. J Clin Oncol. (2025) 43:3355–68. doi: 10.1200/JCO-25-00436 40929627

[17] National Cancer Institute . SEER Cancer Stat Facts: Pancreatic Cancer. Bethesda, MD: National Cancer Institute. Available online at: https://seer.cancer.gov/statfacts/html/pancreas.html (Accessed 2026).

[18] Elshami M Pawar OS Winter JM . Cure probabilities of patients with pancreatic ductal adenocarcinoma in the United States: a Surveillance, Epidemiology, and End Results-based analysis. Surgery. (2026) 195:110223. doi: 10.1016/j.surg.2026.110223 42102533

[19] Crippa S Malleo G Langella S Ricci C Casciani F Belfiori G . Cure probabilities after resection of pancreatic ductal adenocarcinoma: a multi-institutional analysis of 2554 patients. Ann Surg. (2024) 280:999–1005. doi: 10.1097/SLA.0000000000006166 38048334

[20] Rhim AD Mirek ET Aiello NM Maitra A Bailey JM McAllister F . EMT and dissemination precede pancreatic tumor formation. Cell. (2012) 148:349–61. doi: 10.1016/j.cell.2011.11.025 22265420 PMC3266542

[21] Moffitt RA Marayati R Flate EL Volmar KE Loeza SG Hoadley KA . Virtual microdissection identifies distinct tumor- and stroma-specific subtypes of pancreatic ductal adenocarcinoma. Nat Genet. (2015) 47:1168–78. doi: 10.1038/ng.3398 26343385 PMC4912058

[22] O'Kane GM Grünwald BT Jang GH Masoomian M Picardo S Grant RC . GATA6 expression distinguishes classical and basal-like subtypes in advanced pancreatic cancer. Clin Cancer Res. (2020) 26:4901–10. doi: 10.1158/1078-0432.CCR-19-3724 32156747

[23] Elyada E Bolisetty M Laise P Flynn WF Courtois ET Burkhart RA . Cross-species single-cell analysis of pancreatic ductal adenocarcinoma reveals antigen-presenting cancer-associated fibroblasts. Cancer Discov. (2019) 9:1102–23. doi: 10.1158/2159-8290.CD-19-0094 31197017 PMC6727976

[24] Provenzano PP Cuevas C Chang AE Goel VK Von Hoff DD Hingorani SR . Enzymatic targeting of the stroma ablates physical barriers to treatment of pancreatic ductal adenocarcinoma. Cancer Cell. (2012) 21:418–29. doi: 10.1016/j.ccr.2012.01.007 22439937 PMC3371414

[25] Olive KP Jacobetz MA Davidson CJ Gopinathan A McIntyre D Honess D . Inhibition of Hedgehog signaling enhances delivery of chemotherapy in a mouse model of pancreatic cancer. Science. (2009) 324:1457–61. doi: 10.1126/science.1171362 19460966 PMC2998180

[26] Feig C Jones JO Kraman M Wells RJ Deonarine A Chan DS . Targeting CXCL12 from FAP-expressing carcinoma-associated fibroblasts synergizes with anti-PD-L1 immunotherapy in pancreatic cancer. Proc Natl Acad Sci USA. (2013) 110:20212–7. doi: 10.1073/pnas.1320318110 24277834 PMC3864274

[27] Clark CE Hingorani SR Mick R Combs C Tuveson DA Vonderheide RH . Dynamics of the immune reaction to pancreatic cancer from inception to invasion. Cancer Res. (2007) 67:9518–27. doi: 10.1158/0008-5472.CAN-07-0175 17909062

[28] Joo I Lee JM Lee ES Son JY Lee DH Ahn SJ . Preoperative CT classification of the resectability of pancreatic cancer: interobserver agreement. Radiology. (2019) 293:343–9. doi: 10.1148/radiol.2019190422 31502935

[29] Loizou L Duran CV Axelsson E Andersson M Keussen I Strinnholm J . Radiological assessment of local resectability status in patients with pancreatic cancer: interreader agreement and reader performance in two different classification systems. Eur J Radiol. (2018) 106:69–76. doi: 10.1016/j.ejrad.2018.06.014 30150053

[30] van Dongen JC Versteijne E Bonsing BA Mieog JSD de Hingh IHJT Festen S . The yield of staging laparoscopy for resectable and borderline resectable pancreatic cancer in the PREOPANC randomized controlled trial. Eur J Surg Oncol. (2023) 49:811–7. doi: 10.1016/j.ejso.2022.12.011 36585300

[31] Buchs NC Frossard JL Rosset A Chilcott M Koutny-Fong P Chassot G . Vascular invasion in pancreatic cancer: evaluation of endoscopic ultrasonography, computed tomography, ultrasonography, and angiography. Swiss Med Wkly. (2007) 137:286–91. doi: 10.4414/smw.2007.11701 17594541

[32] Tran Cao HS Balachandran A Wang H Nogueras-González GM Bailey CE Lee JE . Radiographic tumor-vein interface as a predictor of intraoperative, pathologic, and oncologic outcomes in resectable and borderline resectable pancreatic cancer. J Gastrointest Surg. (2014) 18:269–78. doi: 10.1007/s11605-013-2374-3 24129826 PMC4016045

[33] Raman SP Reddy S Weiss MJ Manos LL Cameron JL Zheng L . Impact of the time interval between MDCT imaging and surgery on the accuracy of identifying metastatic disease in patients with pancreatic cancer. AJR Am J Roentgenol. (2015) 204:W37–42. doi: 10.2214/AJR.13.12439 25539271 PMC4872713

[34] Sanjeevi S Ivanics T Lundell L Kartalis N Andrén-Sandberg Å Blomberg J . Impact of delay between imaging and treatment in patients with potentially curable pancreatic cancer. Br J Surg. (2016) 103:267–75. doi: 10.1002/bjs.10046 26572509

[35] Groot VP Rezaee N Wu W Cameron JL Fishman EK Hruban RH . Patterns, timing, and predictors of recurrence following pancreatectomy for pancreatic ductal adenocarcinoma. Ann Surg. (2018) 267:936–45. doi: 10.1097/SLA.0000000000002234 28338509

[36] Honselmann KC Pergolini I Fernández-del Castillo C Deshpande V Ting D Taylor MS . Timing but not patterns of recurrence is different between node-negative and node-positive resected pancreatic cancer. Ann Surg. (2020) 272:357–65. doi: 10.1097/SLA.0000000000003123 32675550 PMC6639153

[37] Blackford AL Canto MI Klein AP Hruban RH Goggins M . Recent trends in the incidence and survival of stage 1A pancreatic cancer: a Surveillance, Epidemiology, and End Results analysis. J Natl Cancer Inst. (2020) 112:1162–9. doi: 10.1093/jnci/djaa004 31958122 PMC7669234

[38] Crippa S Pergolini I Javed AA Honselmann KC Weiss MJ Di Salvo F . Implications of perineural invasion on disease recurrence and survival after pancreatectomy for pancreatic head ductal adenocarcinoma. Ann Surg. (2022) 276:378–85. doi: 10.1097/SLA.0000000000004464 33086324

[39] Qin T Li J Xiao Y Wang X Gong M Wang Q . Honokiol suppresses perineural invasion of pancreatic cancer by inhibiting SMAD2/3 signaling. Front Oncol. (2021) 11:728583. doi: 10.3389/fonc.2021.728583 34671554 PMC8521150

[40] Hata T Mizuma M Motoi F Ohtsuka H Nakagawa K Morikawa T . Prognostic impact of postoperative circulating tumor DNA as a molecular minimal residual disease marker in patients with pancreatic cancer undergoing surgical resection. J Hepatobiliary Pancreat Sci. (2023) 30:815–24. doi: 10.1002/jhbp.1282 36408698

[41] Belfiori G Crippa S Aleotti F Pagnanelli M Tamburrino D Gasparini G . Long-term survivors after upfront resection for pancreatic ductal adenocarcinoma: an actual 5-year analysis of disease-specific and post-recurrence survival. Ann Surg Oncol. (2021) 28:8249–60. doi: 10.1245/s10434-021-10401-7 34258720

[42] Latenstein AEJ van Roessel S van der Geest LGM Bonsing BA Dejong CHC Groot Koerkamp B . Conditional survival after resection for pancreatic cancer: a population-based study and prediction model. Ann Surg Oncol. (2020) 27:2516–24. doi: 10.1245/s10434-020-08235-w 32052299 PMC7311496

[43] Hyman DW Almerey T Mujkanovic A Hammons I Tice M Stauffer JA . Comparing post-operative outcomes of pancreaticoduodenectomy for pancreatic ductal adenocarcinoma: neoadjuvant therapy versus surgery first approach. Am Surg. (2022) 88:1868–74. doi: 10.1177/00031348221087900 35465681

[44] Latenstein AEJ Blonk L Tjahjadi NS de Jong N Busch OR de Hingh IHJT . Long-term quality of life and exocrine and endocrine insufficiency after pancreatic surgery: a multicenter, cross-sectional study. HPB (Oxford). (2021) 23:1722–31. doi: 10.1016/j.hpb.2021.04.012 34001452

[45] Wang J Ma R Churilov L Eleftheriou P Nikfarjam M Christophi C . The cost of perioperative complications following pancreaticoduodenectomy: a systematic review. Pancreatology. (2018) 18:208–20. doi: 10.1016/j.pan.2017.12.008 29331217

[46] Jajja MR Mustansir F Nadeem SO Lovasik BP Blair CM Sarmiento JM . Counting the cost: financial implications of complications following pancreaticoduodenectomy. HPB (Oxford). (2022) 24:1177–85. doi: 10.1016/j.hpb.2021.12.012 35078715

[47] US Bureau of Labor Statistics . Consumer Price Index — Medical Care (CUUR0000SAM). Washington, DC: BLS (2026). Available online at: https://www.bls.gov/cpi/ (Accessed October 05, 2026).

[48] DaCosta Byfield S Nash Smyth E Mytelka D Bowman L Teitelbaum A . Healthcare costs, treatment patterns, and resource utilization among pancreatic cancer patients in a managed care population. J Med Econ. (2013) 16:1379–86. doi: 10.3111/13696998.2013.848208 24074258

[49] Stukalin I Ahmed NS Fundytus AM Qian AS Coward S Kaplan GG . Trends and projections in national United States health care spending for gastrointestinal malignancies (1996-2030). Gastroenterology. (2022) 162:1098–110. doi: 10.1053/j.gastro.2021.12.244 34922947 PMC8986994

[50] American Cancer Society . Cancer Facts & Figures 2026. Atlanta, GA: American Cancer Society (2026).

[51] Tramontano AC Chen Y Watson TR Eckel A Sheehan DF Peters MLB . Pancreatic cancer treatment costs, including patient liability, by phase of care and treatment modality, 2000-2013. Med (Baltimore). (2019) 98:e18082. doi: 10.1097/MD.0000000000018082 31804317 PMC6919520

[52] Versteijne E Suker M Groothuis K Akkermans-Vogelaar JM Besselink MG Bonsing BA . Preoperative chemoradiotherapy versus immediate surgery for resectable and borderline resectable pancreatic cancer: results of the Dutch randomized phase III PREOPANC trial. J Clin Oncol. (2020) 38:1763–73. doi: 10.1200/JCO.19.02274 32105518 PMC8265386

[53] Hammel P Huguet F van Laethem JL Goldstein D Glimelius B Artru P . Effect of chemoradiotherapy vs chemotherapy on survival in patients with locally advanced pancreatic cancer controlled after 4 months of gemcitabine with or without erlotinib: the LAP07 randomized clinical trial. JAMA. (2016) 315:1844–53. doi: 10.1001/jama.2016.4324 27139057

[54] Katz MHG Shi Q Meyers J Herman JM Chuong M Wolpin BM . Efficacy of preoperative mFOLFIRINOX vs mFOLFIRINOX plus hypofractionated radiotherapy for borderline resectable adenocarcinoma of the pancreas: the A021501 phase 2 randomized clinical trial. JAMA Oncol. (2022) 8:1263–70. doi: 10.1001/jamaoncol.2022.2319 35834226 PMC9284408

[55] Bergquist JR Ivanics T Shubert CR Habermann EB Smoot RL Kendrick ML . Type of resection (Whipple vs. distal) does not affect the national failure to provide post-resection adjuvant chemotherapy in localized pancreatic cancer. Ann Surg Oncol. (2017) 24:1731–8. doi: 10.1245/s10434-016-5762-6 28070725

[56] Tang HJ Qian D Sondak VK Stachura S Lin J . A modified p53 enhances apoptosis in sarcoma cell lines mediated by doxorubicin. Br J Cancer. (2004) 90:1285–92. doi: 10.1038/sj.bjc.6601653 15026814 PMC2409661

[57] Bouvet M Bold RJ Lee J Evans DB Abbruzzese JL Chiao PJ . Adenovirus-mediated wild-type p53 tumor suppressor gene therapy induces apoptosis and suppresses growth of human pancreatic cancer. Ann Surg Oncol. (1998) 5:681–8. doi: 10.1007/BF02303477 9869513

[58] Ghaneh P Greenhalf W Humphreys M Wilson D Zumstein L Lemoine NR . Adenovirus-mediated transfer of p53 and p16(INK4a) results in pancreatic cancer regression *in vitro* and *in vivo*. Gene Ther. (2001) 8:199–208. doi: 10.1038/sj.gt.3301394 11313791

[59] Tesnière A Schlemmer F Boige V Kepp O Martins I Ghiringhelli F . Immunogenic death of colon cancer cells treated with oxaliplatin. Oncogene. (2010) 29:482–91. doi: 10.1038/onc.2009.356 19881547

[60] Mahadevan KK McAndrews KM LeBleu VS Yang S Lyu H Li B . KRAS(G12D) inhibition reprograms the microenvironment of early and advanced pancreatic cancer to promote FAS-mediated killing by CD8+ T cells. Cancer Cell. (2023) 41:1606–20. doi: 10.1016/j.ccell.2023.07.002 37625401 PMC10785700

[61] Suzuki E Kapoor V Jassar AS Kaiser LR Albelda SM . Gemcitabine selectively eliminates splenic Gr-1+/CD11b+ myeloid suppressor cells in tumor-bearing animals and enhances antitumor immune activity. Clin Cancer Res. (2005) 11:6713–21. doi: 10.1158/1078-0432.CCR-05-0883 16166452

[62] Hingorani SR Zheng L Bullock AJ Seery TE Harris WP Sigal DS . HALO 202: randomized phase II study of PEGPH20 plus nab-paclitaxel/gemcitabine versus nab-paclitaxel/gemcitabine in patients with untreated, metastatic pancreatic ductal adenocarcinoma. J Clin Oncol. (2018) 36:359–66. doi: 10.1200/JCO.2017.74.9564 29232172

[63] Van Cutsem E Tempero MA Sigal D Oh DY Fazio N Macarulla T . Randomized phase III trial of pegvorhyaluronidase alfa with nab-paclitaxel plus gemcitabine for patients with hyaluronan-high metastatic pancreatic adenocarcinoma. J Clin Oncol. (2020) 38:3185–94. doi: 10.1200/JCO.20.00590 32706635 PMC7499614

[64] Özdemir BC Pentcheva-Hoang T Carstens JL Zheng X Wu CC Simpson TR . Depletion of carcinoma-associated fibroblasts and fibrosis induces immunosuppression and accelerates pancreas cancer with reduced survival. Cancer Cell. (2014) 25:719–34. doi: 10.1016/j.ccr.2014.04.005 24856586 PMC4180632

[65] Lin M Liang S Wang X Liang Y Zhang M Chen J . Cryoablation combined with allogenic natural killer cell immunotherapy improves the curative effect in patients with advanced hepatocellular cancer. Oncotarget. (2017) 8:81967–77. doi: 10.18632/oncotarget.17804 29137237 PMC5669863

[66] Garnier J Turrini O Chretien AS Olive D . Local ablative therapy associated with immunotherapy in locally advanced pancreatic cancer: a solution to overcome the double trouble? A comprehensive review. J Clin Med. (2022) 11:1948. doi: 10.3390/jcm11071948 35407555 PMC8999652

[67] Shingleton WB Sewell PE . Percutaneous renal tumor cryoablation with magnetic resonance imaging guidance. J Urol. (2001) 165:773–6. doi: 10.1016/S0022-5347(05)66523-8 11176465

[68] Shingleton WB Sewell PE . Cryoablation of renal tumours in patients with solitary kidneys. BJU Int. (2003) 92:237–9. doi: 10.1046/j.1464-410x.2003.04322.x 12887474

[69] Sewell PE Howard JC Shingleton WB Harrison RB . Interventional magnetic resonance image-guided percutaneous cryoablation of renal tumors. South Med J. (2003) 96:708–10. doi: 10.1097/01.SMJ.0000051766.60936.0E 12940328

[70] Yan Q Hu D Li M Chen Y Wu X Ye Q . The serum microRNA signatures for pancreatic cancer detection and operability evaluation. Front Bioeng Biotechnol. (2020) 8:379. doi: 10.3389/fbioe.2020.00379 32411694 PMC7201024

[71] Buscail L Bournet B Vernejoul F Cambois G Lulka H Hanoun N . First-in-man phase 1 clinical trial of gene therapy for advanced pancreatic cancer: safety, biodistribution, and preliminary clinical findings. Mol Ther. (2015) 23:779–89. doi: 10.1038/mt.2015.1 25586689 PMC4395782

[72] Aguilar LK Shirley LA Chung VM Marsh CL Walker J Coyle W . Gene-mediated cytotoxic immunotherapy as adjuvant to surgery or chemoradiation for pancreatic adenocarcinoma. Cancer Immunol Immunother. (2015) 64:727–36. doi: 10.1007/s00262-015-1679-3 25795132 PMC11029723

[73] Simon R . Optimal two-stage designs for phase II clinical trials. Control Clin Trials. (1989) 10:1–10. doi: 10.1016/0197-2456(89)90015-9 2702835

[74] Niu L Chen J He L Liao M Yuan Y Zeng J . Combination treatment with comprehensive cryoablation and immunotherapy in metastatic pancreatic cancer. Pancreas. (2013) 42:1143–9. doi: 10.1097/MPA.0b013e3182965dde 23899940

[75] Luo XM Niu LZ Chen JB Xu KC . Advances in cryoablation for pancreatic cancer. World J Gastroenterol. (2016) 22:790–800. doi: 10.3748/wjg.v22.i2.790 26811625 PMC4716077

[76] Li Y Lam SSK Gao Y Shore E Anderson DW Hode T . Enhancing the immunotherapeutic effect by IP-001 and irreversible electroporation in mouse oligometastatic models of pancreatic adenocarcinoma. Ann Surg Oncol. (2025) 32:2786–98. doi: 10.1245/s10434-024-16742-3 39739260

[77] Irreversible Electroporation and Pembrolizumab in Locally Advanced Pancreatic Cancer (Immune Correlative Study). Clinicaltrials.Gov Identifier NCT06378047. Available online at: https://clinicaltrials.gov/study/NCT06378047 (Accessed October 05, 2026).

[78] Genentech Inc BioNTech SE . A Study of Autogene Cevumeran (RO7198457) in Combination With Atezolizumab and Mfolfirinox Versus Mfolfirinox in Resected Pancreatic Ductal Adenocarcinoma (IMCODE003). Clinicaltrials.Gov Identifier NCT05968326. Available online at: https://clinicaltrials.gov/study/NCT05968326 (Accessed October 05, 2026).

